# Loss-of-Function of a Tomato Receptor-Like Kinase Impairs Male Fertility and Induces Parthenocarpic Fruit Set

**DOI:** 10.3389/fpls.2019.00403

**Published:** 2019-04-16

**Authors:** Hitomi Takei, Yoshihito Shinozaki, Ryoichi Yano, Sachiko Kashojiya, Michel Hernould, Christian Chevalier, Hiroshi Ezura, Tohru Ariizumi

**Affiliations:** ^1^Graduate School of Life and Environmental Sciences, University of Tsukuba, Tsukuba, Japan; ^2^Japan Society for the Promotion of Science (JSPS), Kôjimachi, Japan; ^3^UMR1332 BFP, Institut National de la Recherche Agronomique (INRA), Villenave-d’Ornon, France; ^4^UMR1332 BFP, University of Bordeaux, Bordeaux, France; ^5^Tsukuba-Plant Innovation Research Center, University of Tsukuba, Tsukuba, Japan

**Keywords:** *Solanum lycopersicum*, fruit set, male sterility, *in situ* hybridization, next generation sequencing, gene mapping

## Abstract

Parthenocarpy arises when an ovary develops into fruit without pollination/fertilization. The mechanisms involved in genetic parthenocarpy have attracted attention because of their potential application in plant breeding and also for their elucidation of the mechanisms involved in early fruit development. We have isolated and characterized a novel *small parthenocarpic fruit and flower* (*spff*) mutant in the tomato (*Solanum lycopersicum*) cultivar Micro-Tom. This plant showed both vegetative and reproductive phenotypes including dwarfism of floral organs, male sterility, delayed flowering, altered axillary shoot development, and parthenocarpic production of small fruits. Genome-wide single nucleotide polymorphism array analysis coupled with mapping-by-sequencing using next generation sequencing-based high-throughput approaches resulted in the identification of a candidate locus responsible for the *spff* mutant phenotype. Subsequent linkage analysis and RNA interference-based silencing indicated that these phenotypes were caused by a loss-of-function mutation of a single gene (*Solyc04g077010*), which encodes a receptor-like protein kinase that was expressed in vascular bundles in young buds. Cytological and transcriptomic analyses suggested that parthenocarpy in the *spff* mutant was associated with enlarged ovarian cells and with elevated expression of the gibberellin metabolism gene, *GA20ox1*. Taken together, our results suggest a role for *Solyc04g077010* in male organ development and indicate that loss of this receptor-like protein kinase activity could result in parthenocarpy.

## Introduction

The flower-to-fruit transition, also known as “fruit set,” corresponds to a major developmental shift that transforms an ovary into a fruit (Gillaspy et al., 1993). This genetically programmed process is coordinated by a complex network of signaling pathways that are activated by interacting endogenous and exogenous cues, although the genetic and molecular factors that control the flower-to-fruit transition remain poorly understood (Ariizumi et al., 2013). The development of parthenocarpic fruit has been observed under some conditions; this pollination-independent seedless fruit can arise when fertilization is inefficient, mainly due to male sterility. Some naturally occurring tomato genetic parthenocarpy has been identified, and these parthenocarpic mutants have been designated *pat*, *pat-2*, and *Pat-k/SlAGL6* (Shinozaki and Ezura, 2016; Klap et al., 2017; Takisawa et al., 2018). The *pat* mutant is characterized by short anthers, partial male sterility, and the production of small fruits (Mazzucato et al., 1998). The locus of the gene responsible for *pat* phenotypes was narrowed down to chromosome 3 (Beraldi et al., 2004). In addition, the gene encoding *SlGA20ox1*, the key enzyme for gibberellin (GA) accumulation in the pollinated tomato ovary, is highly expressed in *pat* ovaries; this is likely to activate GA metabolism and increase GA levels in the unpollinated ovaries, thus triggering parthenocarpy (Olimpieri et al., 2007). The *pat-2* phenotype appears to be caused by a recessive mutation at a single locus on chromosome 4, in a gene encoding a zinc finger homeodomain protein (Nunome, 2016); GA also accumulates at high levels in unpollinated *pat-2* ovaries (Fos et al., 2000). Furthermore, it has been shown that fruit set initiation through both pollination-dependent and -independent processes occurs concomitantly with the down-regulation of a family of floral homeotic MADS-box genes, which regulate floral organ identities (Wang et al., 2009; Tang et al., 2015). Indeed, the loss of function of several MADS-box genes can cause tomato parthenocarpy. For instance, the loss of function of *tomato MADS-box 29*, *tomato MADS-box 5*, and *DEFENCIENS*/*TOMATO APETALA3*/*STAMENLESS* result in parthenocarpy, together with abnormal stamen differentiation (Pnueli et al., 1994; Ampomah-Dwamena et al., 2002; Mazzucato et al., 2008; Quinet et al., 2014; Okabe et al., 2019). Moreover, parthenocarpy was induced in tomatoes that were genetically transformed in order to inhibit stamen development at an early stage of differentiation via the expression of the *BARNASE* ribonuclease gene under a stamen-specific promoter (Medina et al., 2013). Although the mechanisms underlying the role of the stamen in parthenocarpy have not yet been fully characterized, it has been hypothesized that stamens could counteract fruit set initiation before pollination in tomato plants, and this may be associated in part with elevated levels of GA (Okabe et al., 2019).

Flowers and fruits are considered to represent sink organs because their development requires high level of nutrients such as sucrose, as a carbon source (Osorio et al., 2014). The vasculature within flowers, fruits, and their pedicels is therefore of major importance because it transports nutrients and water to these organs (Rančić et al., 2010). XYLEM INTERMIXED WITH PHLOEM1 (XIP1) is one of the proteins with a key role in the organization of vasculature in *Arabidopsis* (Shiu and Bleecker, 2001). This protein is a leucine-rich repeat receptor-like kinase (RLK) that belongs to a large family with at least 216 members encoded in the *Arabidopsis* genome. A loss of XIP1 resulted in modification of vascular bundle organization and abnormal lignification of phloem cells, transforming them to xylem cells (Bryan et al., 2012).

To identify key regulators of parthenocarpy, the present study characterized a novel tomato parthenocarpic mutant known as *small parthenocarpic fruit and flower* (*spff*), which was isolated from a population where mutations were introduced via exposure to γ-ray irradiation (Saito et al., 2011). The *spff* mutant exhibits small flower formation, male sterility, and increased transcription of *GA20ox1* in young ovaries. Furthermore, a rapid high-throughput approach followed by functional validation using RNA interference (RNAi) resulted in the identification of a gene encoding a novel RLK protein.

## Materials and Methods

### Plant Material and Growth Conditions

Tomato wild-type (WT) plants, *Solanum lycopersicum* “Ailsa-Craig” and “Micro-Tom,” and *spff* mutant plants were grown in pots and irrigated daily with Otsuka first and Otsuka second fertilizer solutions under greenhouse conditions in Tsukuba, Japan. The greenhouse was maintained at the ambient temperature and light photoperiod in July and August. WT *S*. *lycopersicum* “Micro-Tom” and *spff* plants for RNA sequence (RNA-seq) analysis, RNAi experiments, and histological analyses were grown in rockwool and irrigated daily with Otsuka first and Otsuka second under vertical farm conditions at 25°C with a 16/8 h light/dark cycle.

### Histological Analysis

Histological analysis of flower tissues was processed as described by Hao et al. (2017). Wax-embedded floral buds were cut into 10-μm cross-sections, layered onto glass slides, and dried overnight at 42°C. The cell size and the number of cell layers were evaluated and the significance of group differences were statistically analyzed using Student *t* test.

### Pollen Number and Germination Assay

Pollens were obtained from anthers at the anthesis stage and germinated in 1 mL of pollen germination medium (0.52 M sucrose, 1.6 mM boric Acid, 1 mM CaCl_2_, 1 mM Ca(NO_3_)_2_, 1 mM MgSO_4_, and 0.01 mM Tris–HCl, pH 7.0). After incubation for 16 h at room temperature, pollen grains were observed under a light microscope. The pollen germination ratio was calculated by dividing the number of germinated pollen (in which the size of the pollen tube is twice or more the diameter of the pollen grain) by the total number of pollen grains; this was defined as the number of pollens observed within one microscopic field. The determinations were made for three replicate biological experiments.

### High-Density Genetic Mapping

For genetic mapping by an Infinium assay (Illumina) using the SolCAP single-nucleotide polymorphism (SNP) array^1^, an F_2_ population was derived from a cross between the *spff* mutant (Micro-Tom background) and WT plants (Ailsa-Craig background). Genomic DNA of 44 F_2_ plants (43 with *spff* mutant phenotypes and one with the WT phenotype), together with F_1_, WT Micro-Tom and parental plants of each genotype, was extracted from fresh leaves using Maxwell 16 DNA purification kits, according to the manufacturer’s protocol (Promega). A total of 48 DNA samples were then used for the SolCAP analysis, using the method described by Sim et al. (2012). Of the 7600 markers analyzed, 1956 markers showed polymorphisms that distinguished between Micro-Tom and Ailsa-Craig; these were used for genotyping. SNPs were obtained from the Kazusa Marker Database^2^. For the linkage analysis, we examined the genotypes at the position 59,966,064 bp on chromosome 4 with the tomInf4732 SNP marker, with sequences of AAGCTT and AAGATT in Micro-Tom and Ailsa-Craig, respectively. Each genotype was discriminated using the primers listed in Supplementary Table S1, followed by restriction digestion with *Hin*d III for 8 h at 37°C.

### Mapping-By-Sequencing

For further fine mapping based on the mapping-by-sequencing approach (Abe et al., 2012; Garcia et al., 2016), an F_2_ population was constructed by crossing the *spff* mutant and WT, in the Micro-Tom background (Supplementary Figure S1). Genomic DNA was extracted from fresh leaves of F_2_ plants that exhibited the *spff* mutant phenotype, as described above. The same amount of extracted DNA from 20 individual plants was pooled and sequenced by 100 bp paired-end sequencing (HiSeq 2000; Illumina). Mutation or variant information was obtained using the Bowtie2-Samtools-GATK (Genome Analysis Tool Kit) pipeline (Li et al., 2009; McKenna et al., 2010; Langmead and Salzberg, 2012). Briefly, Illumina short reads were aligned onto the tomato genome reference SL2.40 by Bowtie2 version 2.2.1^3^ with default parameters. Mutations or variants including SNPs or insertion-deletions (Indels) were then detected by GATK version 3.5 (McKenna et al., 2010). SNPs and Indels that might cause non-synonymous amino acid substitution, a premature stop codon, or frameshift were identified using HaplotypeCaller, as described previously (McKenna et al., 2010; Pulungan et al., 2018). Allele frequency datasets were also obtained using GATK. Because the Micro-Tom cultivar is not inbred and relatively many intra-cultivar variations are present between individuals, we subtracted such intra-cultivar variants from the SNP/Indel datasets using next generation sequencing datasets of several WT Micro-Tom individuals (Pulungan et al., 2018). Candidate genes with a high SNP/Indel index and reliable read numbers (≥10) were then identified. In this analysis, the SNP/Indel index was calculated as the proportion of sequenced reads that included mutant allele SNPs or Indels, in relation to the WT allele.

### Linkage Analysis of the *spff* Locus

The *spff* mutant was backcrossed four times with Micro-Tom WT in order to purify the responsible mutation and finally obtain BC_4_F_2_ plants (Supplementary Figure S1). Linkage analysis was performed using DNA extracted from F_2_, BC_2_F_2_, BC_3_F_2_, and BC_4_F_2_ populations (Supplementary Table S1). Genomic DNA was extracted by DNeasy Miniprep kit (QIAGEN) and amplified by PCR with *TaKaRa Ex Taq* (TAKARA) and the primer set shown in Supplementary Table S2. The PCR products were purified by the Illustra ExoStar kit (GE Healthcare) and then sent to Eurofins Genomics for sequencing.

### Construction of the RNAi Plasmid

The RNAi construct was designed using Gateway technology (Invitrogen). Total RNA was extracted from WT ovaries using the RNeasy Plant Mini Kit (QIAGEN), followed by the removal of genomic DNA using RNA Clean & Concentrator (ZYMO RESEARCH). cDNAs were then synthesized using the SuperScript VILO cDNA Synthesis Kit (Thermo Fisher Scientific). A 521 bp fragment of the *Solyc04g077010* transcript was amplified using the KOD Plus kit (TOYOBO); the cDNA was used as the template, and SlXIPRNAiF1 and SlXIPRNAiR1 were the primers (Supplementary Table S2). The amplicon was then cloned into the donor pBI-sense, antisense-GW vector (INPLANTA INNOVATIONS INC., Japan), allowing expression under the control of the constitutive 35S promoter. The resulting plasmid was introduced into WT Micro-Tom by *Agrobacterium*-mediated transformation using *A. tumefaciens* GV2260 (Sun et al., 2006). Transgenic lines were selected on Murashige and Skoog (MS) agar plates containing kanamycin (100 mg L^-1^).

### RNA Sequencing

Ovaries were collected from flowers at anthesis, separated into three replicates (15–17 ovaries in each replicate) and ground in liquid nitrogen. Total RNA extraction from the ovaries and subsequent cDNA synthesis were performed as described above. Genome-wide RNA expression levels were analyzed by HiSeq (Illumina) with 100 bp single-end reads. The raw reads were subjected to quality filtering before employing the TopHat2-Cufflinks pipeline to calculate the number of reads and calculate expression levels using the reads per kb of transcript per million mapped reads (RPKM), as described previously (Yano et al., 2018). Comprehensive data were analyzed using multiple *t* tests (*p* < 0.05), followed by the Bonferroni correction method, with false discovery rate analysis. Genes with mean RPKM values of ≥1 (three replicates) were considered to be expressed. Genes were considered differentially expressed if the log2 fold ratios were ≥ 1.0 or ≤-1.0, with false discovery rate adjusted *p* values (*q* values) of < 0.05.

### Expression Analysis by Quantitative Reverse Transcription PCR (qRT-PCR) and RT-PCR

For qRT-PCR analysis, the leaves were ground to a fine powder in liquid nitrogen. Total RNA extraction from the samples and subsequent reverse transcription reactions were performed as described above. PCRs were carried out by the CFX96 system (Bio-Rad), using the SYBR *Premix Ex Taq* kit (TaKaRa) and the appropriate gene-specific primers (Supplementary Table S2) according to previously described procedures (Shinozaki et al., 2015). Technical triplicates were performed for each sample, with biological triplicates. The expression levels were calculated using the delta-delta CT method (Pfaffl, 2001), with normalization to the expression of the reference gene, *SAND* (Expósito-Rodríguez et al., 2008). For RT-PCR analysis, cDNA synthesis was performed as described above and equal amount of cDNA was used as templete to observe level of *SPFF* mRNA in various tissues.

### *In situ* Hybridization

The riboprobes used to detect *spff* transcripts were made from a 775 bp fragment amplified from tomato root cDNAs by PCR using the ishF2-ishR1 primer set. The PCR product was used for subsequent PCR using the ishT7F2-ishR1 primer set for sense, and the ishF2-ishT7R1 primer set for antisense, riboprobes; this introduced the T7 RNA polymerase promoter at the 5′ and 3′ ends, respectively. Labeled riboprobes were synthesized by *in vitro* transcription in the presence of digoxigenin-UTP (DIG RNA Labeling kit, SP6/T7; Roche) and used for *in situ* hybridization. The plant tissue processing and *in situ* RNA hybridization experiments were performed following the protocol described by Sicard et al. (2008). Primer sequences used in this study are shown in Supplementary Table S2. For the comparative analysis between WT and *spff* mutant, both WT and *spff* mutant samples were mounted on the same glass slides to allow the direct comparison under the same condition.

## Results

### Identification of the Single Recessive Parthenocarpic *spff* Mutant

**FIGURE 1:**
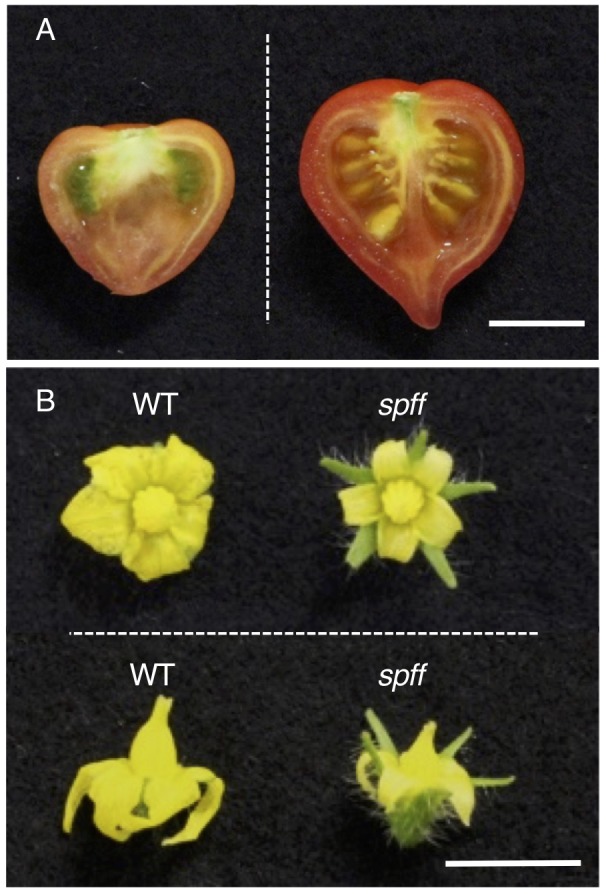
Parthenocarpic fruit and small flower production in *spff* mutant cultivar Micro-Tom. **(A)** Representative fruit of the *spff* mutant. Spontaneously obtained parthenocarpic fruit (left) and seeded fruit from manual pollination of the flower with WT pollen (right) are shown. **(B)** Comparison of WT and *spff* flowers at anthesis stage. Bars are 1 cm.

A visual screening of tomato M_3_ populations obtained after γ-ray irradiation-induced mutagenesis in the genetic background of Micro-Tom, a dwarf and rapid growth variety (Matsukura et al., 2007; Saito et al., 2011), resulted in the isolation of a mutant line (TOMJPG4121) that produced small seedless parthenocarpic fruit (Figure 1A). These plants also produced smaller flowers than the WT plant, particularly due to their narrower petals and shorter anthers (Figure 1B). We therefore called this line the *spff* mutant. Although the *spff* mutant did not produce seeded fruits by practical self-pollination, crossing WT pollen to the *spff* stigma did result in seeded fruits (Figure 1A); these F_1_ seeds germinated normally, suggesting that *spff* is male-sterile, with the ovary retaining substantial fertility. Furthermore, all of the resulting six F_1_ plants exhibited normal flower morphology, with no evidence of parthenocarpic ability, indicating that these mutant phenotypes were recessive. Thirty-three out of 109 F_2_ progenies obtained through crossing with the WT cultivar Micro-Tom, and 43 out of 186 F_2_ progenies obtained through crossing with the WT cultivar Ailsa-Craig, exhibited the *spff* mutant flower morphology and parthenocarpy phenotypes (Table 1 and Supplementary Figure S2). These segregation ratios corresponded to the expected 3:1 for a single recessive gene (Chi-squared = 1.62 for Micro-Tom and 0.35 for Ailsa-Craig background at *p* < 0.05 ). These data suggested the presence of a monogenic recessive mutation in the *spff* line. In the *spff* and WT cultivar Micro-Tom or Ailsa-Craig F_2_ populations, anthesis of the first flower was delayed in the plants with the *spff* phenotype for 19 or 15 days, respectively, as compared to plants with the WT phenotype (Supplementary Figure S3); this indicated that the flowering delay trait was tightly associated with the *spff* flower morphology and parthenocarpy phenotypes.

**Table 1:** Segregation test of *spff* mutant traits at F_2_ progeny of a self-pollinated F_1_ plant.

Parental genotypes	The number of F_2_ plants analyzed for segregation test			
	Total number of plants	WT phenotypes	*spff* mutant phenotypes	χ^2^ (3:1)^a^
*spff* × Micro-Tom (WT)	109	76	33	1.62 ns
*spff* × Ailsa Craig (WT)	186	143	43	0.35 ns

*The spff mutant phenotypes were evident as small flower and parthenocarpic fruit formation.*

*^a^chi-squared test (p < 0.05). ns, not significant.*

### Characterization of the Pleiotropic Mutant Phenotypes in *spff*

**FIGURE 2:**
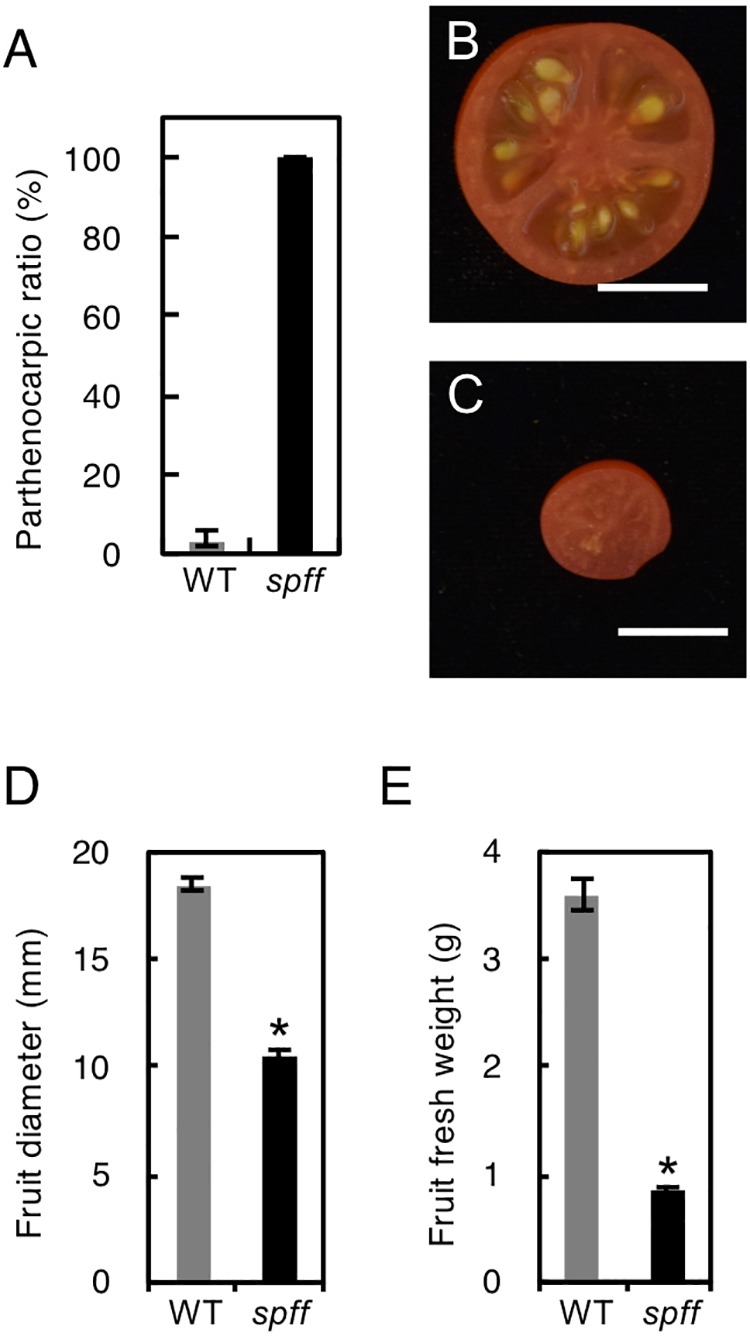
Inflorescence and fruit phenotypes of the *spff* mutant. **(A)** Parthenocarpic ratio in spontaneously produced WT and *spff* fruit. Fruit morphologies in WT **(B)** and *spff* **(C)**. Quantification analyses focused on the fruit diameter **(D)** and fruit fresh weight **(E)**. Bars are 1 cm. At least three biological repetitions were performed and their mean values with SE are shown.

**FIGURE 3:**
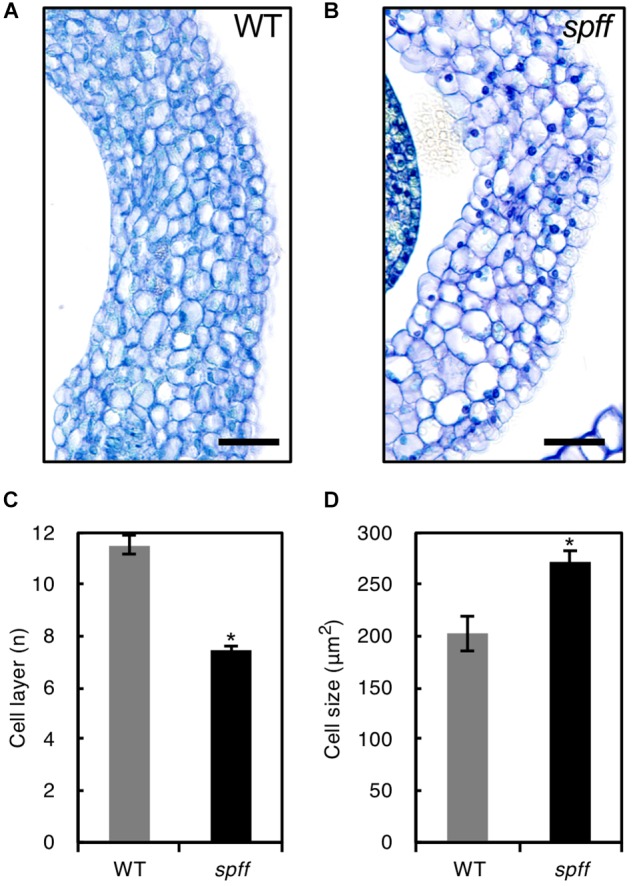
Ovary wall histology in WT and *spff*. Transverse ovary wall sections of WT **(A)** and *spff* mutant **(B)** at anthesis. Number of cell layers **(C)** and cell size **(D)** in WT and *spff* ovary walls. Bars are 30 μm. At least three biological repetitions were performed and their mean values with SE are shown. Asterisks indicate significant difference between WT and *spff* mutant (Student *t* test, *p* < 0.01).

For detailed phenotypic characterizations, the *spff* mutant in the M_3_ population was backcrossed four times with WT cultivar Micro-Tom pollen to reduce mutagen-induced background mutations (Supplementary Figure S1). The resulting BC_4_F_2_ plants that exhibited *spff* phenotypes were analyzed. First, we examined the parthenocarpic phenotype in the *spff* mutant. The *spff* yielded obligate parthenocarpic fruit under spontaneous production, and this was not observed in WT plants (Figure 2A). Compared to the pollinated WT fruits, the *spff* parthenocarpic fruits were smaller and lighter (Figure 2B–E). For cytological characterization of parthenocarpy at the early developmental stage, we prepared cross-sections of the ovaries at anthesis and examined the number of cell layers and cell size within the pericarp (Figure 3). The *spff* mutant cells were significantly larger than the WT cells, by approximately 1.3-fold (WT = 202 ± 17 μm^2^, *spff* = 272 ± 11 μm^2^), and *spff* had fewer cell layers. This suggested that *spff* parthenocarpy was associated with cell enlargement, rather than active cell division.

**FIGURE 4:**
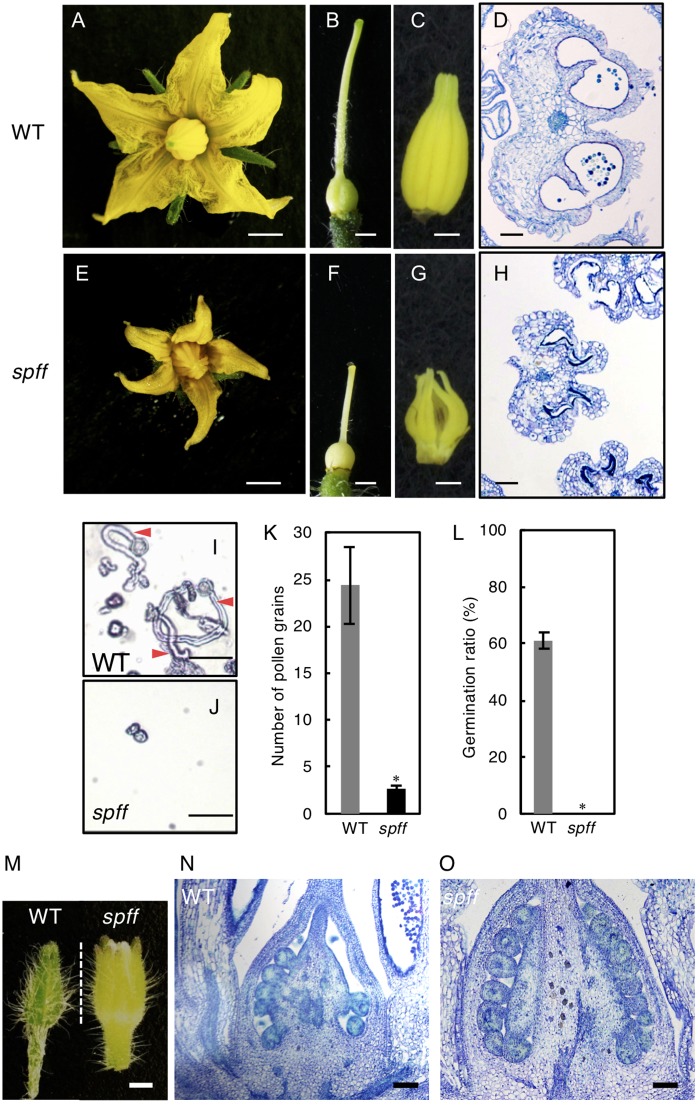
Reproductive organ phenotypes in the *spff* mutant. Morphology of flower **(A,D)**, pistil **(B,E)**, and anthers **(C,F)** in WT **(A–C)** and *spff* **(D–F)** at anthesis. Histological sections of WT **(G)** and *spff* **(H)** anthers at the anthesis stage. **(I)** Pollen tube elongation from WT pollen. Red arrowheads indicate pollen tubes. **(J)** Pollen grains from the *spff* mutant, without elongation of pollen tubes. **(K)** Number of pollen grains in a microscopic field. **(L)** Germination ratio of pollen tubes. **(M)** Appearance of floral bud length of 4 mm. Longitudinal section of ovaries from 4 mm floral buds **(M)** in WT **(N)** and *spff* **(O)**. Bars are 2 mm **(A,E)**; 1 mm **(B,C,F,G)**; 100 μm **(D,H,N,O)**, and 50 μm **(I,J)**. At least three biological repetitions were performed and their mean values with SE are shown. Asterisks indicate significant difference between WT and *spff* mutant (Student *t* test, *p* < 0.01).

Further, the smaller flowers produced in *spff* reflected the presence of smaller constitutive tissues, including the petals, style, and anthers; the clearly defective anther may explain the male-sterility of this mutant (Figure 4A–H). To evaluate the male fertility of *spff*, cross-sections of the WT and *spff* anthers at the anthesis stage were compared. The oval-shaped WT anther locules included pollen grains that showed a germination rate of approximately 60 ± 5% (Figure 4D,I,K,L). In contrast, the *spff* anther locules were shrunken and contained very few pollen grains, which were unable to germinate (Figure 4H,J–L); this indicated that the *spff* mutant was fully male sterile. In addition, histological observations of the *spff* and WT ovaries at the bud length 4 mm indicated the presence of equivalent internal structures, except for their size (Figure 4M–O), consistent with the fact that the *spff* retained substantial female fertility (Table 1). We also found that *spff* affected plant architecture, with an altered pattern of axillary shoot development (Supplementary Figures S4A–C). The lateral branches of *spff* showed increased sympodial growth, in which vegetative and inflorescence stems were more actively developed from the individual first axillary buds, leading to a bushy plant morphology. These data characterizing the phenotypes of *spff* indicated that the mutation conferred pleiotropic effects on both reproductive and vegetative morphology in tomato plants.

**FIGURE 5:**
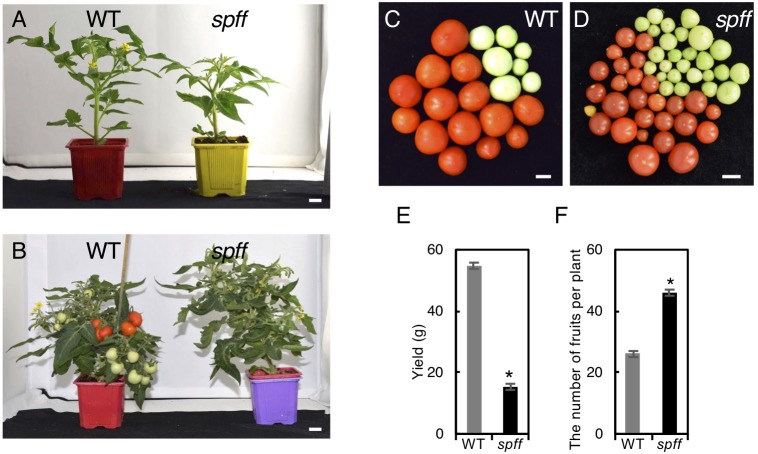
Fruit phenotypes and yield evaluation of the *spff* mutant. **(A)** Plant appearance of WT (left) and *spff* (right) mutant at 40 days and **(B)** 66 days after seed germination. The *spff* grew slowly than WT. Bars are 1 cm. Representative harvested fruit in WT **(C)** and *spff* mutant **(D)** after 112 and 173 days of seed germination, respectively, and their yield of mature red fruits **(E)**. **(F)** The number of total fruit produced in WT and *spff* mutant after 112 and 173 days of seed germination, respectively. At least three biological repetitions were performed and their mean values with SE are shown. Asterisks indicate significant difference between WT and *spff* mutant (Student *t* test, *p* < 0.01).

We next compared yield potential between WT and *spff* mutant. Since *spff* mutant showed significant growth delay compared to WT leading to late fruit production (Figure 5A,B), which made it difficult to conduct comparative yield quantification, WT and *spff* mutant plants were grown in a greenhouse for 112 and 173 days, respectively, until they nearly reached vegetative growth maturation, determining the yield of ripe red fruits as well as the total number of fruits per plant. The yield (total weight) of ripe red fruit in *spff* mutant was reduced to 28 % of WT albeit longer growth period and higher number of fruits per plant, suggesting less impact of its potential for improving yield (Figure 5C–F).

### Identification of the Gene Associated With the *spff* Phenotype

**FIGURE 6:**
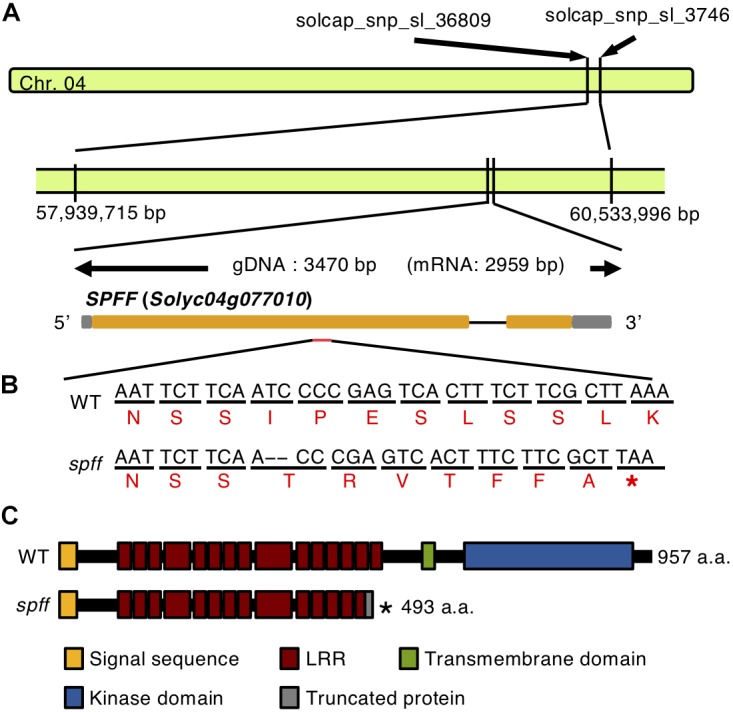
Gene structure of *Solyc04g077010.* **(A)** The location and gene structure of *Solyc04g077010.* Chromosome 4 is indicated by the green bar. The gray boxes indicate the untranslated regions, orange boxes indicate the exons, separated by the introns. **(B)** Nucleotide and amino acid sequences of the WT and the *spff* mutant around the mutation point. The upper black letters indicate nucleotides, and the lower red letters indicate putative amino acids. The 2 bp deletion indicated by hyphens introduces a premature stop codon in the first exon in the *spff* mutant. **(C)** The putative amino acid length and domain structure of the RLK encoded by *Solyc04g077010*, in WT and the *spff* mutant. In **B**, **C** ^∗^indicate the stop codon.

The *spff* mutation was mapped using an F_2_ population obtained by crossing *spff* mutants (Micro-Tom background) with WT plants (Ailsa-Craig background) by an Illumina SNP Infinium analysis with the SolCAP array (Sim et al., 2012). We generated in total of 186 F_2_ plants consisting of 143 plants showing WT phenotypes and of 43 plants showing *spff* mutant phenotypes (Table 1). Genotyping of 43 plants with the *spff* mutant phenotype using 1956 SNP markers pointed to a 2.6 Mbp region flanked by two SNP markers (solcap_snp_sl_36809 and solcap_snp_sl_3746) on chromosome 4. Based on the tomato genome release SL2.40, these two markers flanked locations at positions 57,939,715 bp and 60,553,996 bp, respectively (Figure 6A and Supplementary Figure S5). No other possible candidate loci were detected, consistent with the fact that the *spff* phenotypes reflected a monogenic recessive mutation (Table 1). According to the Kazusa Marker Database^3^ (based on SL2.40), this candidate region included 267 protein-coding genes. The tomInf4732 SNP, which discriminated between Ailsa-Craig and Micro-Tom alleles within the candidate region using primer set F4-R4 (Supplementary Table S2), was used to further genotype 73 F_2_ plants. These included 43 plants with *spff* phenotypes and 30 with WT phenotypes, allowing us to narrow down the region of interest to 2.0 Mbp, which included 205 genes.

We next employed mapping-by-sequencing (Abe et al., 2012; Garcia et al., 2016) of an F_2_ population derived by crossing *spff* with WT in a Micro-Tom background. DNA from 20 individual mutant phenotype F_2_ plants was sequenced by Illumina HiSeq, and cleaned reads were mapped onto the cultivar Micro-Tom reference genome; polymorphisms were substituted against the cultivar Heinz reference genome version SL2.40 (Kobayashi et al., 2014). The Bowtie2-Samtools-GATK pipeline identified and calculated the frequencies of potential *spff*-specific SNPs and Indels. This analysis identified 77 mutant homozygous SNPs and Indels within the region narrowed down by SNP Infinium analysis (Supplementary Table S3). These 77 mutations were present in the coding regions of 46 genes, which were considered to represent candidate genes for the *spff* phenotype (Supplementary Table S4). Five of these candidates (*Solyc04g076020*, *Solyc04g076100*, *Solyc04g076250*, *Solyc04g076320*, and *Solyc04g077010*) were chosen for further linkage analysis. These were selected because of their relatively high expression levels in flowers and fruits, according to tomato eFP browser (Winter et al., 2007; The Tomato Genome Consortium, 2012), and because of the predicted impact of the mutation on the encoded protein. Their linkages with the *spff* phenotypes were analyzed using marker-based approaches at F_2_ and backcrossed populations listed in Supplementary Table S1 with the five primer sets shown in Supplementary Table S2. The F18-R18 marker for a 2 bp deletion in the *Solyc04g077010* gene (Figure 6B), which encodes an RLK, showed perfect segregation with the *spff* phenotypes. All of the 83 mutant-phenotype plants, and none of the 80 non-parthenocarpic plants, were homozygous for this mutation; the non-parthenocarpic plants were either heterozygous or azygous for this mutation, while four other mutations were not perfectly linked with the *spff* phenotypes (Supplementary Table S5). We realized that the gene model of *Solyc04g077010* in the tomato gene annotation ITAG2.3/SL2.40 differed from the latest ITAG3.2/SL3.0^4^, in which *Solyc04g077010* consists of two exons spanning 2871 bp and encoding 957 amino acids. The mutation identified in the present study was located in the first exon and led to a frame shift, which introduced a premature stop codon at position 494 and therefore generated a truncated protein composed of 493 amino acids (Figure 6C). RLK proteins are structurally characterized by three conserved domains: a receptor domain containing a varying number of leucine-rich repeats; a transmembrane domain; and a kinase domain that transduces the downstream signal via autophosphorylation (Shiu and Bleecker, 2001). The RLK protein encoded by *Solyc04g077010* harbors a single transmembrane domain between amino acids 505 and 524. This suggested that the mutation would cause a loss-of-function of this protein, thus resulting in the *spff* mutant phenotypes.

**FIGURE 7:**
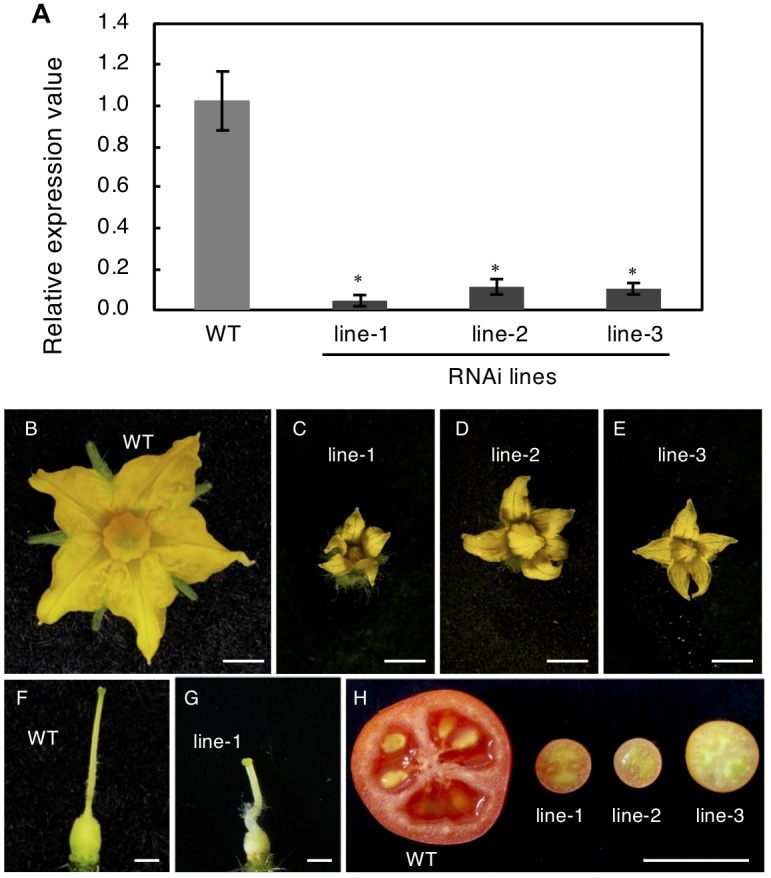
Knock-Down of *SPFF* gene expression in RNAi transgenic tomato lines. **(A)** Expression analysis of *SPFF* gene in the leaves of WT and RNAi lines. The floral morphology of WT **(B)** and the RNAi plants line-1 **(C)**, line-2 **(D)** and line-3 **(E)**. The pistil morphology of WT **(F)** and the RNAi plant line-1 **(G)**. **(H)** Pollinated WT (left) and parthenocarpic fruit in the RNAi lines (right). Bars are 2 mm **(B–E)**, 1 mm **(F,G)** and 1 cm **(H)**. At least three biological repetitions were performed and their mean values with SE are shown. Asterisks indicate significant difference between WT and RNAi plant (Student *t* test, *p* < 0.01).

To confirm this, RNAi was used to reduce *Solyc04g077010* expression. The RNAi vector targeted the first exon of this gene, which encoded a highly specific receptor domain that was confirmed to be unlikely conserved in other tomato genes encoding RLK proteins by the BLAST search. The RNAi vector was introduced into Micro-Tom plants and three transgenic lines were obtained; these showed significantly reduced mRNA expression of the target protein (Figure 7A). These three independent transgenic plants showed resemblance to *spff* phenotypes such as producing small flowers and fruits with parthenocarpy (Figure 7B–H). Further, those RNAi showed complete male sterility, while pollination of WT pollen gave rise to mature viable seeds as observed in *spff* mutant. These analyses demonstrated that the *spff* phenotypes resulted from a loss-of-function of this RLK protein-encoding gene.

### Vasculature-Specific Expression of *SPFF* Gene in Flower Receptacle

The *in silico* expression profile obtained by RNA-seq and RT-PCR analyses (Winter et al., 2007; The Tomato Genome Consortium, 2012) revealed that *Solyc04g077010* was expressed in various plant organs, including roots, leaves, buds, and flowers (Supplementary Figures S7A, S8). Previously published transcriptome data (Ezura et al., 2017) indicated that this gene was expressed in floral organs both before and after anthesis, and transcripts were observed in individual floral organs including the ovary/pistil, anther, petal, and sepal, with the highest expression observed in the ovary/pistil at 1 day before anthesis (Supplementary Figure S7B). Interestingly, a spatio-temporal analysis of the transcriptome of developing tomato fruits (Fernandez-Pozo et al., 2017; Shinozaki et al., 2018b) revealed vasculature-specific expression of *Solyc04g077010* in the fruit pericarp throughout development (Supplementary Figure S7C). Consistent with this, predominant expression of this gene was also found in fruit internal tissues, columella and placenta (Supplementary Figure S7D), with a high abundance in thick vascular bundles.

**FIGURE 8:**
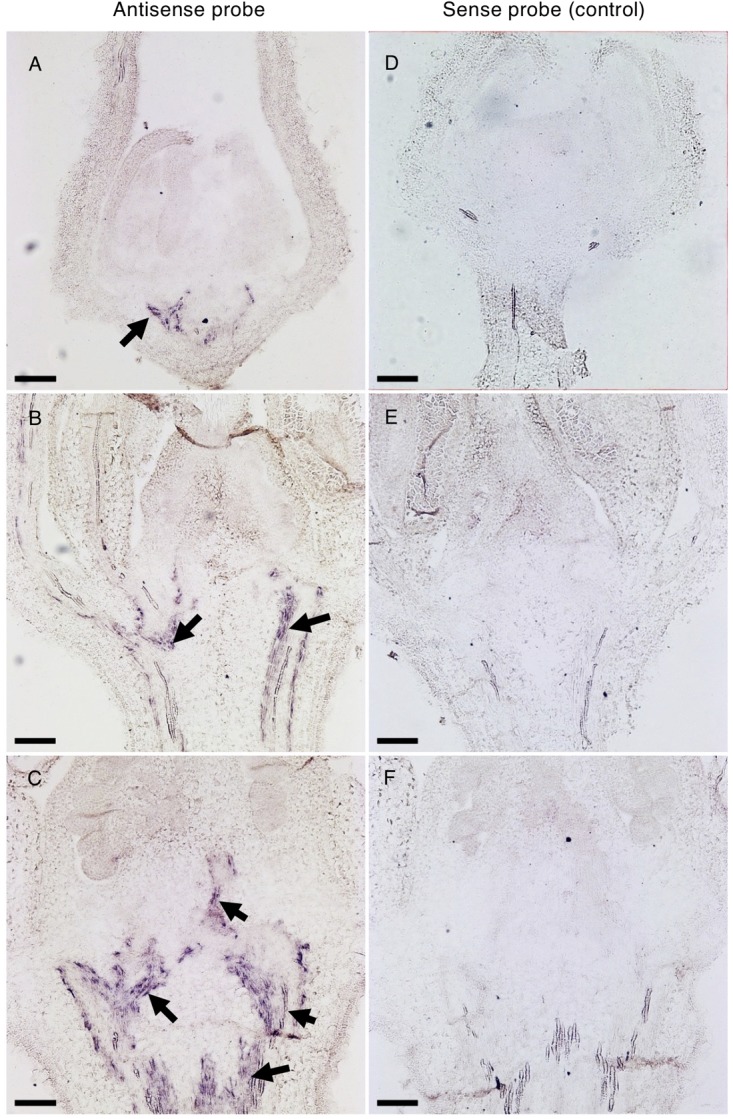
*In situ* hybridization of *Solyc04g077010* transcripts in developing buds. Distribution of *Solyc04g077010* expression was detected by an antisense probe **(A–C)** and a low background signal was detected by a control sense probe **(D–F)** in 1.1 mm **(A,D)**, 2.9 mm **(B,E)**, and 4.5 mm **(C,F)** buds. Black arrows indicate signals detected in vasculature bundles and their distribution in the receptacle, pedicel, and/or columella. Bars are 100 μm.

To unravel the spatio-temporal expression pattern of *Solyc04g077010* during flower development, *in situ* mRNA hybridization was performed in WT floral buds at different stages of development. In the early developing 1.1 mm bud, the transcript signal was exclusively observed in the vasculature tissues of the receptacle (Figure 8A,D). As development proceeded, the *SPFF* transcripts were also detected in the vasculature of the pedicel (2.9 mm bud) (Figure 8B,E), and in the vasculature of the columella tissue (4.5 mm bud) (Figure 8C,F). We also observed reduced *SPFF* transcripts in receptacle and leaves of *spff* compared to WT (Supplementary Figure S6A), indicating that the *spff* mutation influences both transcript abundance and protein function.

### *Solyc04g077010* Mutation May Affect Hormonal Regulation at the Transcriptional Level

To obtain insights into the molecular mechanisms underlying parthenocarpy in the *spff* mutant plant, the ovarian transcriptome at the anthesis stage, corresponding to flower-to-fruit transition, was compared to that of WT plants. Our RNA-seq analysis identified a total of 25 differentially expressed genes; 13 of these were significantly up-regulated in *spff* plants (log2 fold-change > 1) and 12 were significantly down-regulated (log2 fold-change < -1) (*q* values < 0.05 for the comparison with WT, Supplementary Table S6). Notably, the up-regulated genes in the *spff* ovary included *SlGA20ox1* (*Solyc03g006880*), which encodes a key GA biosynthetic enzyme that is induced by pollination and is also highly expressed during parthenocarpy in the *pat* mutant (Olimpieri et al., 2007; Serrani et al., 2007b). In *spff*, the expression level of *SlGA20ox1* was > 10-fold of that observed in the WT plant. This result suggested that GA is involved in the parthenocarpic early transition from flower to fruit exhibited by the *spff* mutant. To gain further insights into this, we compared our differentially expressed genes with previously published transcriptomic data obtained from GA-treated and -untreated unfertilized ovaries (Tang et al., 2015). One of our 13 up-regulated genes (*SlGA20ox1*) and three of our 12 down-regulated genes [*Solyc02g078150* (Plant-specific domain TIGR01615 family protein), *Solyc12g094620* (catalase), and *Solyc05g005150* (F-box/Kelch repeat-containing F-box family protein)] were found in the list of genes that were up- and down-regulated by GA treatment, respectively.

### Flower Receptacle Development Is Not Likely to Be Affected in the *spff* Mutant

A database BLASTP search showed that the protein encoded by XYLEM INTERMIXTED WITH PHLOEM1 (XIP1) is the closest homolog to tomato *SPFF*, with 63% amino acid identity (*E*-value 0, score 1153 bits, and 77% positives) with the *Arabidopsis* counterpart (GenBank accession no. BAC42540.1). *Arabidopsis xip1* loss-of-function mutants showed excessive anthocyanin accumulation in the leaves and severe defects in plant growth, while fertility was not affected (Bryan et al., 2012). Here, the *spff* mutant did not show excessive anthocyanin accumulation in the leaves and showed severe male sterility (Figure 4 and Supplementary Figure S4D). Nevertheless, the fact that the *xip1* mutants altered plant vascular development, represented by intermixed xylem with phloem, suggests a similar function for the *SPFF* protein, whose expression was indeed localized to vasculature in the fruit and inflorescence tissues (Figure 8 and Supplementary Figure S7C). To unravel this, we compared xylem-phloem distribution patterns between WT and *spff* mutant receptacles. Cross-sections of receptacle were stained with Safranin O and Astra blue to visualize lignified (seen as red) and unlignified (seen as blue) tissues. Supplementary Figure S9 shows that the stained receptacle cross-sections did not reveal significant xylem-phloem intermixing in the *spff* mutant.

## Discussion

### The Gene Associated With the *spff* Phenotype Encodes a Putative RLK Involved in Flower and Fruit Development

This study aimed to identify and characterize the gene underlying a newly isolated tomato mutant, named *spff*, which showed parthenocarpy and floral organ dwarfism as its major phenotypes (Figure 1–4). A high-throughput approach combining high-density genetic mapping (Supplementary Figure S5) and mapping-by-sequencing, followed by conventional genetic linkage analysis (Supplementary Tables S3–S5), allowed the rapid identification of a potential causal mutation in a gene located on chromosome 4, *Solyc04g077010* (Figure 6). This gene encodes a potential RLK that appeared to be mainly expressed in the receptacle of young floral buds (Figure 8 and Supplementary Figure S6). A 2 bp deletion mutation was identified, which introduced a premature stop codon that leads to the production of a truncated RLK protein (Figure 6) as well as to reduced transcript abundance (Supplementary Figure S6). Using RNAi approach, we confirmed that the *spff* phenotypes could be reproduced by silencing *Solyc04g077010* (Figure 7), and thus concluded that this is the causative gene for the *spff* mutant.

The *Solyc04g077010* homolog in *Arabidopsis*, *xip1*, was reported to be involved in vascular bundle differentiation (Bryan et al., 2012). The *xip1* mutant shows aberrant xylem-like cells within the phloem in inflorescence stems. Although *Solyc04g077010* appeared to be expressed in close vicinity to the vascular bundle (Figure 8 and Supplementary Figures S6, S7), xylem-like cells were not present within the phloem (Supplementary Figure S9). Moreover, fertility was not affected in *Arabidopsis xip1* mutant plants, where the inflorescence stems are shorter than those of the Col-0 accession plants, and the cotyledons and rosette leaves show a purple color, indicative of anthocyanin accumulation. Since these phenotypes were not observed in the present *spff* mutant (Figure 1, 4 and Supplementary Figure S4), *Solyc04g077010* does not seem to be a functionally conserved ortholog of *XIP1*. It is more likely to be a novel gene that has possibly acquired a specific function in tomato, although further analyses are needed to confirm this functional dissimilarity with the *Arabidopsis XIP1* gene.

### Hypothesis for How the *spff* Mutant Induces Parthenocarpy

Parthenocarpy can mimic the molecular mechanisms underlying pollination-dependent ovary growth (Li et al., 2014). Fruit set initiation and parthenocarpy are regulated by complex hormone networks. Molecular genetic studies of many mutants/genotypes and transcriptome analyses of early fruit development have suggested that parthenocarpy is in part induced through a hierarchical scheme of temporal regulation by multiple hormones, initiated by the accumulation of auxin; this induces intense cell division, with the subsequent induction of GA metabolism triggering active cell expansion (Martí et al., 2007; Serrani et al., 2007a, 2008). Thus, GA should act as the downstream signal and cell expansion most likely plays a crucial role for fruit set initiation in tomato (Serrani et al., 2008; Shinozaki et al., 2015). The present study revealed that the *spff* mutant exhibited higher levels of *GA20ox1* than WT plants (Supplementary Table S6); this is one of the key factors involved in GA biosynthesis in tomato ovaries (Olimpieri et al., 2007; Serrani et al., 2007b). Further, three GA-down regulated genes (*Solyc02g078150*, *Solyc12g094620*, and *Solyc05g005150*) were found in the list of differentially expressed genes identified by the RNA-seq analysis in the unfertilized ovary of *spff* mutant (Supplementary Table S6). In addition, the small parthenocarpic fruits produced by the *spff* mutant were characterized by enlarged cells, rather than an increased number of cell layers in the ovary pericarp, most likely due to a lack of intense cell division (Figure 3). This was consistent with the characteristics of parthenocarpic fruit induced by increased GA sensitivity (Martí et al., 2007). In contrast, auxin-induced parthenocarpy is associated with intensive cell division in the pericarp, resulting in an increased number of cell layers (Wang et al., 2009). The *spff* mutant also showed reduced pollen fertility (Figure 4I–L), which could reflect an increased GA response (Livne et al., 2015). These results suggest that the RLK encoded by *Solyc04g077010* functions to repress the GA response in reproductive organs, and that *spff* parthenocarpy may result in part from an increased GA response.

Additionally, the association of parthenocarpy with early male organ developmental abnormality has been observed in tomato plants. Mutations or genetic suppressions of MADS-box genes, which inhibit functional stamen development by causing homeotic conversions, can induce parthenocarpy (Pnueli et al., 1994; Ampomah-Dwamena et al., 2002; Mazzucato et al., 2008; Quinet et al., 2014; Okabe et al., 2019). Furthermore, the over-accumulation of *BARNASE* mRNA under a stamen-specific promoter triggers early anther ablation and parthenocarpy (Medina et al., 2013), while loss of function of *SEXUAL STERILITY/HYDRA* results in complete male sterility and parthenocarpy (Hao et al., 2017; Rojas-Gracia et al., 2017). Recently, a *tap3* mutant has also been described in which stamens are converted into a carpelloid structure and GA over-accumulates in unfertilized ovaries, most likely due to the overexpression of GA metabolism genes such as *GA20ox1* (Okabe et al., 2019). Taken together with the fact that the *spff* mutant shows male sterility and *GA20ox1* is highly expressed in the unfertilized ovary of the *spff* mutant (Supplementary Table S6), it is possible that parthenocarpy in the *spff* mutant involves increased levels of *GA20ox1* transcripts through the association with male sterility. Since our transcriptome analysis revealed no differential expression of MADS-box genes between WT and *spff* mutants (Supplementary Table S6), and no homeotic conversion phenotypes were observed in the *spff* mutant (Figure 1, 4), the association of floral homeotic genes with the *Solyc04g077010* gene, and the mechanisms involved in *GA20ox1* gene regulation, require further elucidation.

The *in situ* mRNA analysis showed that *Solyc04g077010* was strongly expressed in vascular bundle cells of the floral receptacle and pedicel (Figure 8). Vascular systems in inflorescence stems are important for nutrient and signal transportation during developmental events in the reproductive organs (Rančić et al., 2010). We therefore hypothesize that the RLK encoded by *Solyc04g077010* may be involved in the transportation of molecular substances essential for normal floral organ development, and that loss-of-function mutations of this gene may lead to the disruption of integrity of such a system, which may then cause anther abortion. Since we identified little cytological evidence for structural differences between the vascular bundles observed in WT and *spff* mutant plants (Supplementary Figure S9), future studies are required to investigate this possibility in more detail.

Although the role of RLK family proteins in the regulation of fruit development has yet to be fully delineated, a cell-type specific transcriptome study of tomato ovaries showed that several genes encoding RLKs were enriched in the cluster that is mainly expressed in the funiculus of the developing seed. These included a homolog of the *Arabidopsis HAESA* gene, which is involved in specifying seed abscission zones, suggesting that the tomato homolog may possess a similar function (Pattison et al., 2015). Furthermore, silencing of an invertase inhibitor gene in the *SlINVINH1-RNAi* line, causing increased cell wall invertase activity, was associated with an overall reduction in the transcription of RLK family members in young ovaries, suggesting that RLK may play a role in sensing the modification of cell wall components, thereby regulating downstream gene expression (Ru et al., 2017). Elucidation of RLK activities, including the identification of ligands and kinase domain target proteins, would provide valuable insights into the involvement of RLK proteins in the regulation of fruit development.

## Conclusion

In conclusion, this study identified a novel tomato mutant showing parthenocarpy and this was caused by the loss of function in the gene encoding a receptor kinase gene designated as *SPFF*. The parthenocarpic variety potentially shows improved fruit productivity due to increased fruit set efficiency (Shinozaki et al., 2018a), although the *spff* produced delayed growth, smaller mature fruits and reduced yield compared to WT (Figure 5). Such unfavorable traits render this mutant less attractive for breeding application, but it would be interesting to identify hypomorphic (weaker) alleles of *spff* carrying less detrimental phenotypes through screening from TILLING populations or genome editing approaches (Okabe et al., 2011; Shimatani et al., 2017), and investigate their potentials for impact on breeding application.

## Author Contributions

YS and TA contributed to the mutant screening. HT, YS, RY, and TA contributed to genetic mapping and transcriptomic analysis. HT and YS performed phenotypic characterizations of mutant plants. SK and HT contributed to expression analysis. HT, MH, and CC contributed to histological analysis and *in situ* hybridization assays. HT, YS, MH, CC, HE, and TA wrote the manuscript. All authors reviewed and approved the final manuscript.

## Conflict of Interest Statement

The authors declare that the research was conducted in the absence of any commercial or financial relationships that could be construed as a potential conflict of interest.

## Footnotes

1 http://solcap.msu.edu

2 http://marker.kazusa.or.jp/Tomato/

3 https://solgenomics.net/organism/Solanum_lycopersicum/genome

4 https://solgenomics.net/organism/Solanum_lycopersicum/genome

## Abbreviations

Bc_1_F_2_: Single backcross and second filial generation
Bc_3_F_2_: Backcross three times and second filial generation
Bcip: 5-Bromo-4-chloro-3′-indolyl phosphate
Bsa: Bovine Serum Albumin
F_1_: First filial generation
F_2_: Second filial generation
Faa: Formalin-Acetic acid-Alcohol
Ms: Murashige and Skoog medium
Nbt: Nitroblue Tetrazolium
Pbs: Phosphate buffered saline
Rnai: Rna interference
Sgn: Sol Genomics Network
Snp: Single Nucleotide Polymorphism
Spff: Small Parthenocarpic Fruit and Flower
Sspe: Sodium Chloride-Sodium Phosphate-Edta

## References

[B1] Abe A. Kosugi S. Yoshida K. Natsume S. Takagi H. Kanzaki H. (2012). Genome sequencing reveals agronomically important loci in rice using MutMap. *Nat. Biotechnol.* 30 174–178. 10.1038/nbt.2095 22267009

[B2] Ampomah-Dwamena C. Morris B. A. Sutherland P. Veit B. Yao J.-L. (2002). Down-regulation of *TM29*, a tomato *SEPALLATA* homolog, causes parthenocarpic fruit development and floral reversion. *Plant Physiol.* 130 605–617. 10.1104/pp.005223 12376628 PMC166590

[B3] Ariizumi T. Shinozaki Y. Ezura H. (2013). Genes that influence yield in tomato. *Breed. Sci.* 63 3–13. 10.1270/jsbbs.63.3 23641176 PMC3621442

[B4] Beraldi D. Picarella M. E. Soressi G. P. Mazzucato A. (2004). Fine mapping of the *parthenocarpic fruit* (*pat*) mutation in tomato. *Theor. Appl. Genet.* 108 209–216. 10.1007/s00122-003-1442-6 14564391

[B5] Bryan A. C. Obaidi A. Wierzba M. Tax F. E. (2012). XYLEM INTERMIXED WITH PHLOEM1, a leucine-rich repeat receptor-like kinase required for stem growth and vascular development in *Arabidopsis thaliana*. *Planta* 235 111–122. 10.1007/s00425-011-1489-6 21853254

[B6] Expósito-Rodríguez M. Borges A. A. Borges-Pérez A. Pérez J. A. (2008). Selection of internal control genes for quantitative real-time RT-PCR studies during tomato development process. *BMC Plant Biol.* 8:131. 10.1186/1471-2229-8-131 19102748 PMC2629474

[B7] Ezura K. Ji-Seong K. Mori K. Suzuki Y. Kuhara S. Ariizumi T. (2017). Genome-wide identification of pistil-specific genes expressed during fruit set initiation in tomato (*Solanum lycopersicum*). *PLoS One* 12:e0180003. 10.1371/journal.pone.0180003 28683065 PMC5500324

[B8] Fernandez-Pozo N. Zheng Y. Snyder S. I. Nicolas P. Shinozaki Y. Fei Z. (2017). The tomato expression atlas. *Bioinformatics* 33 2397–2398. 10.1093/bioinformatics/btx190 28379331 PMC5860121

[B9] Fos M. Nuez F. Garcia-Martinez J. L. (2000). The gene *pat-2*, which induces natural parthenocarpy, alters the gibberellin content in unpollinated tomato ovaries. *Plant Physiol.* 122 471–480. 10.1104/pp.122.2.471 10677440 PMC58884

[B10] Garcia V. Bres C. Just D. Fernandez L. Tai F. W. J. Mauxion J.-P. (2016). Rapid identification of causal mutations in tomato EMS populations via mapping-by-sequencing. *Nat. Protoc.* 11 2401–2418. 10.1038/nprot.2016.143 27809315

[B11] Gillaspy G. Ben-David H. Gruissem W. (1993). Fruits: a developmental perspective. *Plant Cell* 5 1439–1451. 10.2307/3869794 12271039 PMC160374

[B12] Hao S. Ariizumi T. Ezura H. (2017). *SEXUAL STERILITY* is essential for both male and female gametogenesis in tomato. *Plant Cell Physiol.* 58 22–34. 10.1093/pcp/pcw214 28082517

[B13] Klap C. Yeshayahou E. Bolger A. M. Arazi T. Gupta S. K. Shabtai S. (2017). Tomato facultative parthenocarpy results from SlAGAMOUS-LIKE 6 loss of function. *Plant Biotechnol. J.* 15 634–647. 10.1111/pbi.12662 27862876 PMC5399002

[B14] Kobayashi M. Nagasaki H. Garcia V. Just D. Bres C. Mauxion J.-P. (2014). Genome-wide analysis of intraspecific DNA polymorphism in “Micro-Tom,” a model cultivar of tomato (*Solanum lycopersicum*). *Plant Cell Physiol.* 55 445–454. 10.1093/pcp/pct181 24319074

[B15] Langmead B. Salzberg S. L. (2012). Fast gapped-read alignment with Bowtie 2. *Nat. Methods* 9 357–359. 10.1038/nmeth.1923 22388286 PMC3322381

[B16] Li H. Handsaker B. Wysoker A. Fennell T. Ruan J. Homer N. (2009). The sequence alignment/map format and SAMtools. *Bioinformatics* 25 2078–2079. 10.1093/bioinformatics/btp352 19505943 PMC2723002

[B17] Li J. Wu Z. Cui L. Zhang T. Guo Q. Xu J. (2014). Transcriptome comparison of global distinctive features between pollination and parthenocarpic fruit set reveals transcriptional phytohormone cross-talk in cucumber (*Cucumis sativus* L.). *Plant Cell Physiol.* 55 1325–1342. 10.1093/pcp/pcu051 24733865

[B18] Livne S. Lor V. S. Nir I. Eliaz N. Aharoni A. Olszewski N. E. (2015). Uncovering DELLA-independent gibberellin responses by characterizing new tomato procera mutants. *Plant Cell* 27 1579–1594. 10.1105/tpc.114.132795 26036254 PMC4498196

[B19] Martí C. Orzáez D. Ellul P. Moreno V. Carbonell J. Granell A. (2007). Silencing of *DELLA* induces facultative parthenocarpy in tomato fruits. *Plant J.* 52 865–876. 10.1111/j.1365-313X.2007.03282.x 17883372

[B20] Matsukura C. Yamaguchi I. Inamura M. Ban Y. Kobayashi Y. Yin Y.-G. (2007). Generation of gamma irradiation-induced mutant lines of the miniature tomato (*Solanum lycopersicum* L.) cultivar *“Micro-Tom.”*. *Plant Biotechnol.* 24 39–44. 10.5511/plantbiotechnology.24.39

[B21] Mazzucato A. Olimpieri I. Siligato F. Picarella M. E. Soressi G. P. (2008). Characterization of genes controlling stamen identity and development in a parthenocarpic tomato mutant indicates a role for the *DEFICIENS* ortholog in the control of fruit set. *Physiol. Plant* 132 526–537. 10.1111/j.1399-3054.2007.01035.x 18334005

[B22] Mazzucato A. Taddei A. R. Soressi G. P. (1998). The *parthenocarpic fruit* (pat) mutant of tomato (*Lycopersicon esculentum* Mill.) sets seedless fruits and has aberrant anther and ovule development. *Development* 125 107–114. 9389668 10.1242/dev.125.1.107

[B23] McKenna A. Hanna M. Banks E. Sivachenko A. Cibulskis K. Kernytsky A. (2010). The genome analysis toolkit: a mapreduce framework for analyzing next-generation DNA sequencing data. *Genome Res.* 20 1297–1303. 10.1101/gr.107524.110 20644199 PMC2928508

[B24] Medina M. Roque E. Pineda B. Cañas L. Rodriguez-Concepcion M. Beltran J. P. (2013). Early anther ablation triggers parthenocarpic fruit development in tomato. *Plant Biotechnol. J.* 11 770–779. 10.1111/pbi.12069 23581527

[B25] Nunome T. (2016). Map-based cloning of tomato parthenocarpic *pat-2* gene. *Regul. Plant Growth Dev.* 51 37–40. 10.18978/jscrp.51.1_37

[B26] Okabe Y. Asamizu E. Saito T. Matsukura C. Ariizumi T. Brès C. (2011). Tomato TILLING technology: development of a reverse genetics tool for the efficient isolation of mutants from Micro-Tom mutant libraries. *Plant Cell Physiol.* 52 1994–2005. 10.1093/pcp/pcr134 21965606 PMC3212723

[B27] Okabe Y. Yamaoka T. Ariizumi T. Ushijima K. Kojima M. Takebayashi Y. (2019). Aberrant stamen development is associated with parthenocarpic fruit set through up-regulation of gibberellin biosynthesis in tomato. *Plant Cell Physiol.* 60 38–51. 10.1093/pcp/pcy184 30192961

[B28] Olimpieri I. Siligato F. Caccia R. Mariotti L. Ceccarelli N. Soressi G. P. (2007). Tomato fruit set driven by pollination or by the *parthenocarpic fruit* allele are mediated by transcriptionally regulated gibberellin biosynthesis. *Planta* 226 877–888. 10.1007/s00425-007-0533-z 17503074

[B29] Osorio S. Ruan Y.-L. Fernie A. R. (2014). An update on source-to-sink carbon partitioning in tomato. *Front. Plant Sci.* 5:516. 10.3389/fpls.2014.00516 25339963 PMC4186278

[B30] Pattison R. J. Csukasi F. Zheng Y. Fei Z. van der Knaap E. Catalá C. (2015). Comprehensive tissue-specific transcriptome analysis reveals distinct regulatory programs during early tomato fruit development. *Plant Physiol.* 168 1684–1701. 10.1104/pp.15.00287 26099271 PMC4528740

[B31] Pfaffl M. W. (2001). A new mathematical model for relative quantification in real-time RT-PCR. *Nucleic Acids Res.* 29:e45 10.1093/nar/29.9.e45 PMC55695 11328886

[B32] Pnueli L. Hareven D. Rounsley S. D. Yanofsky M. F. Lifschitz E. (1994). Isolation of the tomato *AGAMOUS* gene *TAG1* and analysis of its homeotic role in transgenic plants. *Plant Cell* 6 163–173. 10.1105/tpc.6.2.163 7908549 PMC160424

[B33] Pulungan S. I. Yano R. Okabe Y. Ichino T. Kojima M. Takebayashi Y. (2018). *SlLAX1* is required for normal leaf development mediated by balanced adaxial and abaxial pavement cell growth in tomato. *Plant Cell Physiol.* 59 1170–1186. 10.1093/pcp/pcy052 29528453

[B34] Quinet M. Bataille G. Dobrev P. I. Capel C. Gómez P. Capel J. (2014). Transcriptional and hormonal regulation of petal and stamen development by *STAMENLESS*, the tomato (*Solanum lycopersicum* L.) orthologue to the B-class APETALA3 gene. *J. Exp. Bot.* 65 2243–2256. 10.1093/jxb/eru089 24659487 PMC4036497

[B35] Rančić D. Quarrie S. P. Pećinar I. (2010). “Anatomy of tomato fruit and fruit pedicel during fruit development,” in *Microscopy Science, Technology, Applications and Education*, eds Méndez-Vilas A. Díaz J. (Badajoz: Formatex Research Center), 851–861. 10.1111/nph.14433

[B36] Rojas-Gracia P. Roque E. Medina M. Rochina M. Hamza R. Angarita-Díaz M. P. (2017). The parthenocarpic *hydra* mutant reveals a new function for a *SPOROCYTELESS*-like gene in the control of fruit set in tomato. *New Phytol.* 214 1198–1212. 10.1111/nph.14433 28134991

[B37] Ru L. Osorio S. Wang L. Fernie A. R. Patrick J. W. Ruan Y.-L. (2017). Transcriptomic and metabolomics responses to elevated cell wall invertase activity during tomato fruit set. *J. Exp. Bot.* 68 4263–4279. 10.1093/jxb/erx219 28922759 PMC5853505

[B38] Saito T. Ariizumi T. Okabe Y. Asamizu E. Hiwasa-Tanase K. Fukuda N. (2011). TOMATOMA: a novel tomato mutant database distributing Micro-Tom mutant collections. *Plant Cell Physiol.* 52 283–296. 10.1093/pcp/pcr004 21258066 PMC3037083

[B39] Serrani J. C. Fos M. Atarés A. Garcia-Martinez J. L. (2007a). Effect of gibberellin and auxin on parthenocarpic fruit growth induction in the cv Micro-Tom of tomato. *J. Plant Growth Regul.* 26 211–221. 10.1007/s00344-007-9014-7

[B40] Serrani J. C. Sanjuan R. Ruiz-Rivero O. Fos M. Garcia-Martinez J. L. (2007b). Gibberellin regulation of fruit set and growth in tomato. *Plant Physiol.* 145 246–257. 10.1104/pp.107.098335 17660355 PMC1976567

[B41] Serrani J. C. Ruiz-Rivero O. Fos M. García-Martínez J. L. (2008). Auxin-induced fruit-set in tomato is mediated in part by gibberellins. *Plant J.* 56 922–934. 10.1111/j.1365-313X.2008.03654.x 18702668

[B42] Shimatani Z. Kashojiya S. Takayama M. Terada R. Arazoe T. Ishii H. (2017). Targeted base editing in rice and tomato using a CRISPR-Cas9 cytidine deaminase fusion. *Nat. Biotechnol.* 35 441–443. 10.1038/nbt.3833 28346401

[B43] Shinozaki Y. Ezura K. (2016). “Tomato fruit set and its modification using molecular breeding techniques,” in *Functional Genomics and Biotechnology in Solanaceae and Cucurbitaceae Crops*, eds Ezura H. Ariizumi T. Garcia-Mas J. Rose J. (Heidelberg: Springer), 93–112. 10.1007/978-3-662-48535-4_7

[B44] Shinozaki Y. Ezura K. Hu J. Okabe Y. Bénard C. Prodhomme D. (2018a). Identification and functional study of a mild allele of *SlDELLA* gene conferring the potential for improved yield in tomato. *Sci. Rep.* 13:12043. 10.1038/s41598-018-30502-w 30104574 PMC6089951

[B45] Shinozaki Y. Hao S. Kojima M. Sakakibara H. Ozeki-Iida Y. Zheng Y. (2015). Ethylene suppresses tomato (Solanum lycopersicum) fruit set through modification of gibberellin metabolism. *Plant J.* 83 237–251. 10.1111/tpj.12882 25996898

[B46] Shinozaki Y. Nicolas P. Fernandez-Pozo N. Ma Q. Evanich D. J. Shi Y. (2018b). High-resolution spatiotemporal transcriptome mapping of tomato fruit development and ripening. *Nat. Commun.* 9:364. 10.1038/s41467-017-02782-9 29371663 PMC5785480

[B47] Shiu S. H. Bleecker A. B. (2001). Receptor-like kinases from *Arabidopsis* form a monophyletic gene family related to animal receptor kinases. *Proc. Natl. Acad. Sci. U.S.A.* 98 10763–10768. 10.1073/pnas.181141598 11526204 PMC58549

[B48] Sicard A. Petit J. Mouras A. Chevalier C. Hernould M. (2008). Meristem activity during flower and ovule development in tomato is controlled by the mini zinc finger gene *INHIBITOR OF MERISTEM ACTIVITY*. *Plant J.* 55 415–427. 10.1111/j.1365-313X.2008.03520.x 18410478

[B49] Sim S.-C. Durstewitz G. Plieske J. Wieseke R. Ganal M. W. Van Deynze A. (2012). Development of a large SNP genotyping array and generation of high-density genetic maps in tomato. *PLoS One* 7:e40563. 10.1371/journal.pone.0040563 22802968 PMC3393668

[B50] Sun H.-J. Uchii S. Watanabe S. Ezura H. (2006). A highly efficient transformation protocol for Micro-Tom, a model cultivar for tomato functional genomics. *Plant Cell Physiol.* 47 426–431. 10.1093/pcp/pci251 16381658

[B51] Takisawa R. Nakazaki T. Nunome T. Fukuoka H. Kataoka K. Saito H. (2018). The parthenocarpic gene *Pat-k* is generated by a natural mutation of *SlAGL6* affecting fruit development in tomato (*Solanum lycopersicum* L.). *BMC Plant Biol.* 18:72. 10.1186/s12870-018-1285-6 29699487 PMC5921562

[B52] Tang N. Deng W. Hu G. Hu N. Li Z. (2015). Transcriptome profiling reveals the regulatory mechanism underlying pollination dependent and parthenocarpic fruit set mainly mediated by auxin and gibberellin. *PLoS One* 10:e0125355. 10.1371/journal.pone.0125355 25909657 PMC4409352

[B53] The Tomato Genome Consortium (2012). The tomato genome sequence provides insights into fleshy fruit evolution. *Nature* 485 635–641. 10.1038/nature11119 22660326 PMC3378239

[B54] Wang H. Schauer N. Usadel B. Frasse P. Zouine M. Hernould M. (2009). Regulatory features underlying pollination-dependent and -independent tomato fruit set revealed by transcript and primary metabolite profiling. *Plant Cell* 21 1428–1452. 10.1105/tpc.108.060830 19435935 PMC2700536

[B55] Winter D. Vinegar B. Nahal H. Ammar R. Wilson G. V. Provart N. J. (2007). An “Electronic Fluorescent Pictograph” browser for exploring and analyzing large-scale biological data sets. *PLoS One* 2:e718. 10.1371/journal.pone.0000718 17684564 PMC1934936

[B56] Yano R. Nonaka S. Ezura H. (2018). Melonet-DB, a grand RNA-Seq gene expression atlas in melon (*Cucumis melo* L.). *Plant Cell Physiol.* 59:e4. 10.1093/pcp/pcx193 29216378

