# Corrigendum: Loss-of-Function of a Tomato Receptor-Like Kinase Impairs Male Fertility and Induces Parthenocarpic Fruit Set

**DOI:** 10.3389/fpls.2021.672086

**Published:** 2021-04-27

**Authors:** Hitomi Takei, Yoshihito Shinozaki, Ryoichi Yano, Sachiko Kashojiya, Michel Hernould, Christian Chevalier, Hiroshi Ezura, Tohru Ariizumi

**Affiliations:** ^1^Graduate School of Life and Environmental Sciences, University of Tsukuba, Tsukuba, Japan; ^2^Japan Society for the Promotion of Science (JSPS), Kôjimachi, Japan; ^3^UMR1332 BFP, Institut National de la Recherche Agronomique (INRA), Villenave-d'Ornon, France; ^4^UMR1332 BFP, University of Bordeaux, Bordeaux, France; ^5^Tsukuba-Plant Innovation Research Center, University of Tsukuba, Tsukuba, Japan

**Keywords:** *Solanum lycopersicum*, fruit set, male sterility, *in situ* hybridization, next generation sequencing, gene mapping

In the original article, there were mistakes in the legends for Figure 4 and Supplementary Figure 7 as published.

In the first two sentences of the legend to Figure 4, the letters attributed to the different parts of the figure were wrong. The correct legend appears below.

**Figure 4:**
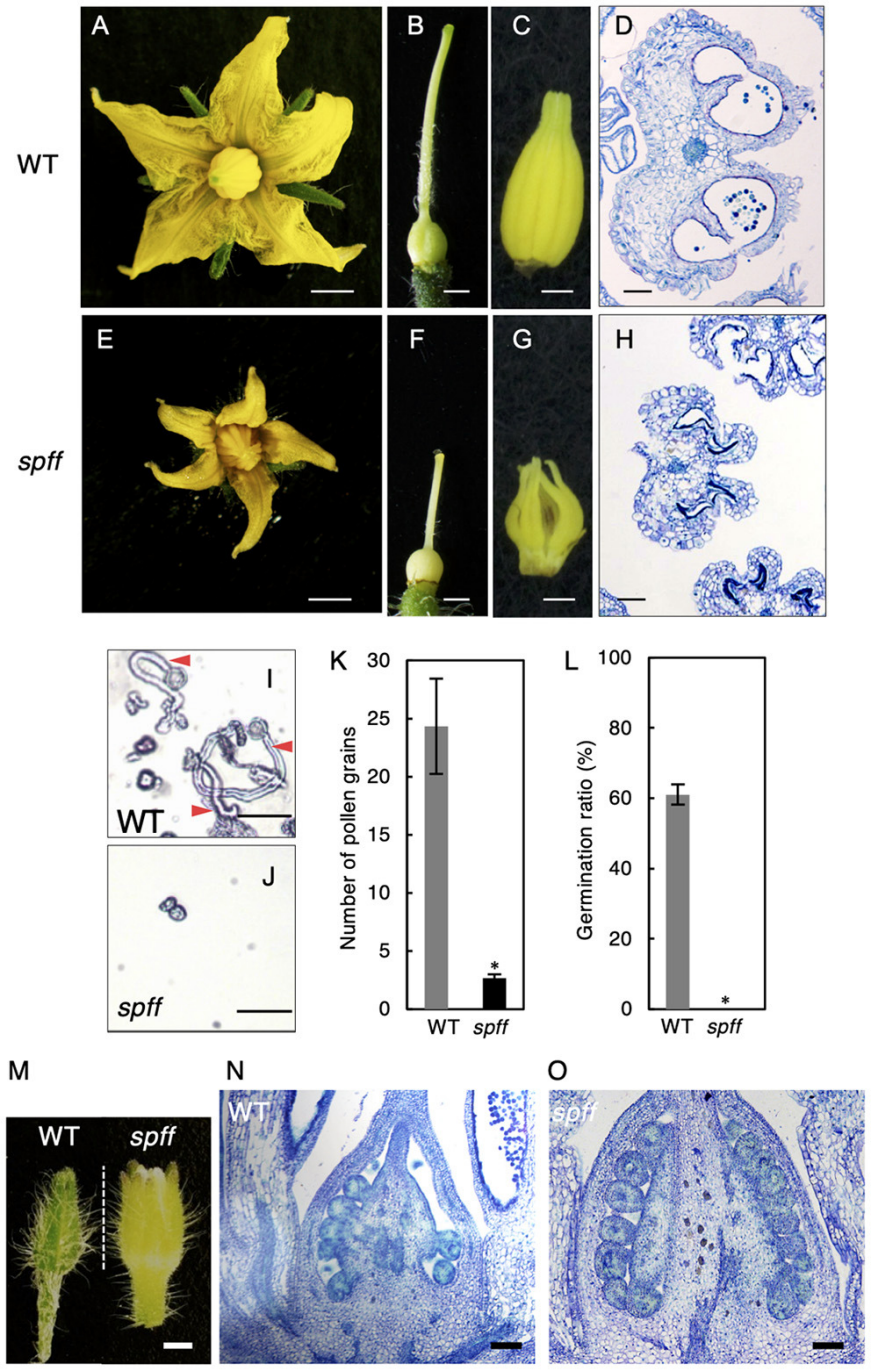
Reproductive organ phenotypes in the *spff* mutant. Morphology of flower **(A,E)**, pistil **(B,F)**, and anthers **(C,G)** in WT **(A-C)** and *spff* **(E-G)** at anthesis. Histological sections of WT **(D)** and *spff* **(H)** anthers at the anthesis stage. **(I)** Pollen tube elongation from WT pollen. Red arrowheads indicate pollen tubes. **(J)** Pollen grains from the *spff* mutant, without elongation of pollen tubes. **(K)** Number of pollen grains in a microscopic field. **(L)** Germination ratio of pollen tubes. **(M)** Appearance of floral bud length of 4 mm. Longitudinal section of ovaries from 4 mm floral buds **(M)** in WT **(N)** and *spff* **(O)**. Bars are 2 mm **(A,E)**; 1 mm **(B,C,F,G)**; 100 μm **(D,H,N,O)**, and 50 μm **(I,J)**. At least three biological repetitions were performed and their mean values with SE are shown. Asterisks indicate significant difference between WT and *spff* mutant (Student *t-*test, *p* < 0.01).

The axis labels in Supplementary Figure 7B were incorrectly formatted and its legend contained some typographical errors. The corrected Supplementary Figure 7 and legend appear below.

**Supplementary Figure 7:**
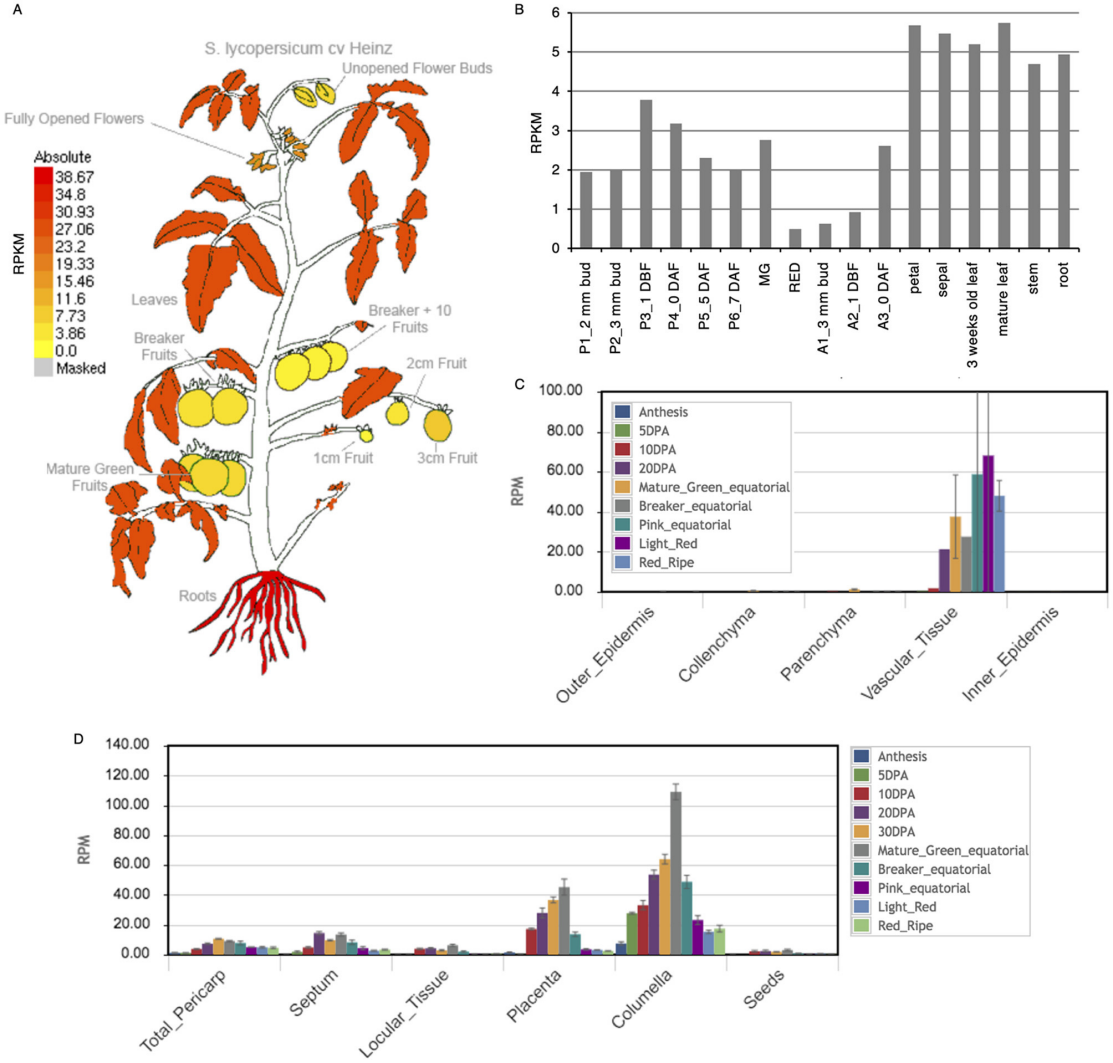
Spatiotemporal expression pattern of *SPFF* in various tomato organs, tissues and cells. **(A)** Expression images of *SPFF*, visualized in Tomato eFP browser (Winter et al., 2007), at tomato plant cv. Heinz 1706 organs (The Tomato Genome Consortium, 2012). **(B)** Expression pattern of *SPFF* in organs and inflorescence tissues of cv. Micro-Tom (Ezura et al., 2017). P1-6, pistil samples; MG, mature green fruits at 33 days after flowering; RED, red fruits at 44 days after flowering; A1-3, anther samples; DBF, days before flowering; DAF, days after flowering. *SPFF* mRNA expression pattern in fruit pericarp cell/tissue **(C)** and fruit tissue **(D)**, visualized in Tomato Expression Atlas (Fernandez-Pozo et al., 2017), in cv. M82 (Shinozaki et al., 2018). DPA, days post anthesis; RPKM, reads per kilobase of exon per million mapped reads; RPM, reads per million mapped reads.

The authors apologize for these errors and state that this does not change the scientific conclusions of the article in any way. The original article has been updated.
