# Supplementary material for: Loss-of-Function of a Tomato Receptor-Like Kinase Impairs Male Fertility and Induces Parthenocarpic Fruit Set
Source: Front Plant Sci. 2019 Apr 16;10:403. doi: 10.3389/fpls.2019.00403 (PMC6477066; doi:10.3389/fpls.2019.00403)
Supplement: Supplementary file 2 [file Presentation_1.pdf]

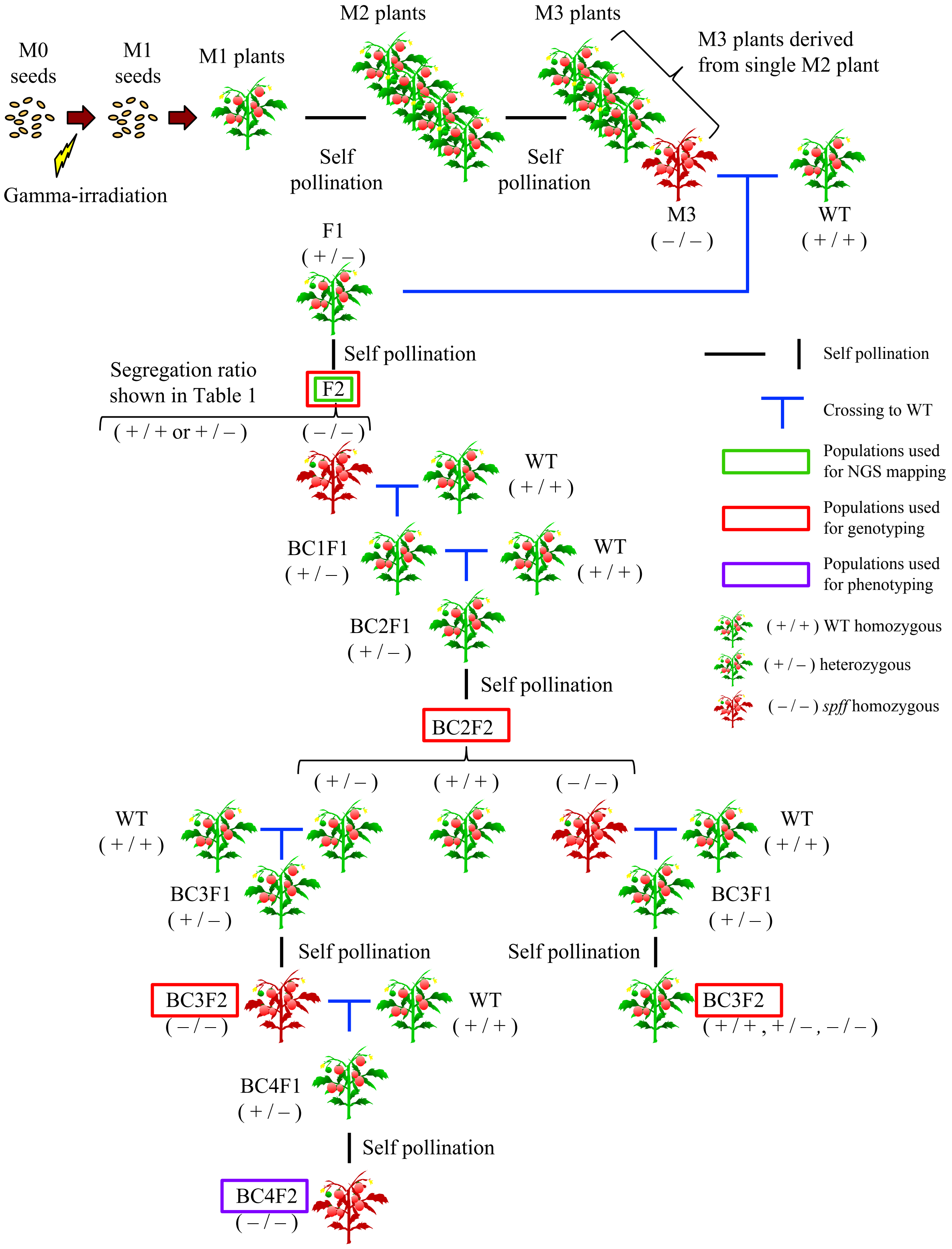

SUPPLEMENTARY FIGURE S1 | Scheme of backcrossing of *spff* mutant to WT. The parthenocarpic mutant named *spff* was originally obtained from gamma-irradiated mutagenized populations of Micro-Tom and the identified M3 *spff* plant was initially backcrossed to WT followed by additional backcrosses to finally generated BC4F2 population to remove background mutations possibly induced by gamma-ray irradiation. Genotyping was determined for either WT (+/+), heterozygous (+/–) or homozygous (–/–) for *spff* mutation using primers listed in **Supplementary Table S2**. BC2F2, BC3F2 and BC4F2 populations were used for genotyping (indicated by red square), while BC4F2 populations were used for phenotyping (indicated by red square).

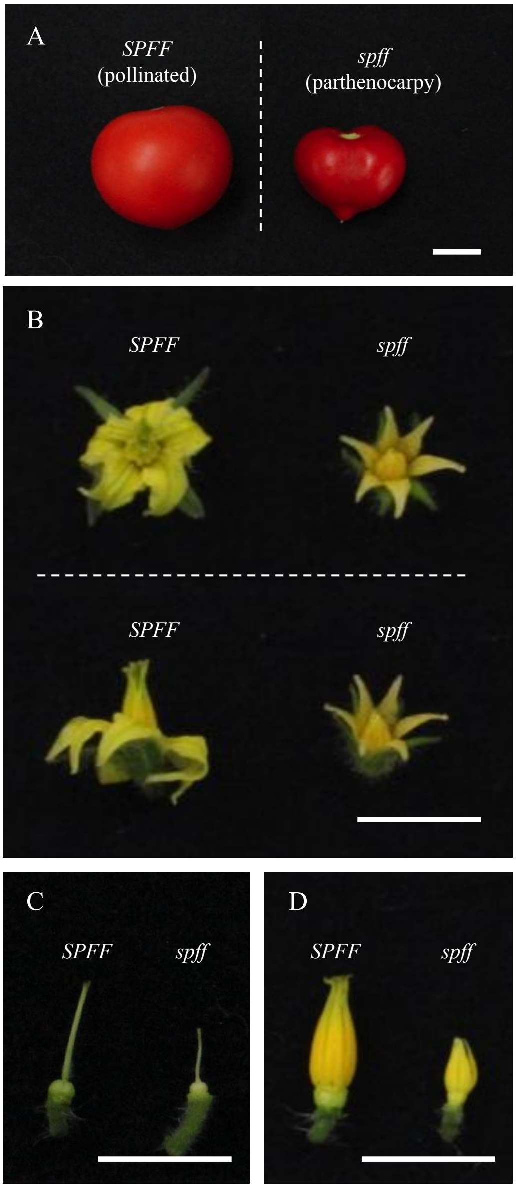

SUPPLEMENTARY FIGURE S2 | Reproductive phenotypes in F_2 plants constructed from a cross between *spff* in Micro-Tom and WT in Ailsa Craig. Representative pictures of fruit **(A)**, flowers **(B)** pistil **(C)**, and anthers **(D)** of *spff* (*spff* homozygous) and *SPFF* (*SPFF* homozygous [WT allele]) plants without dwarf phenotypes conferred by *dwarf* (*d*) mutation. Bars are 1 cm.

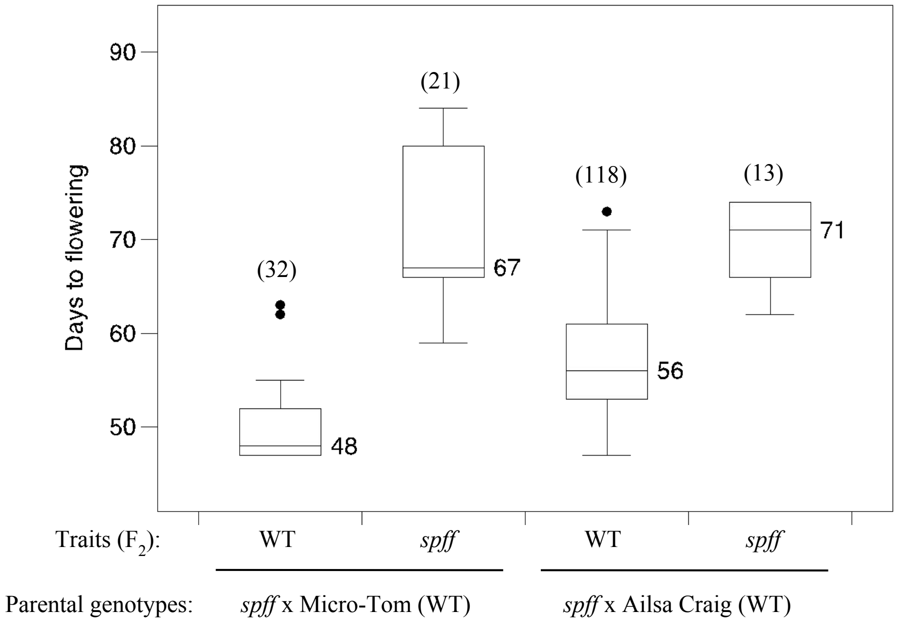

SUPPLEMENTARY FIGURE S3 | Delayed flowering in plants showing *spff* traits at F_2 progeny of a self-pollinated F_1 plant. Boxplots show the interquartile range (IQR) of days to flowering with the median values (indicated by a horizontal line within IQR), data range (vertical line) and outliers (dots). The numbers of observed plants showing WT or *spff* mutant traits are indicated by the values in parentheses.

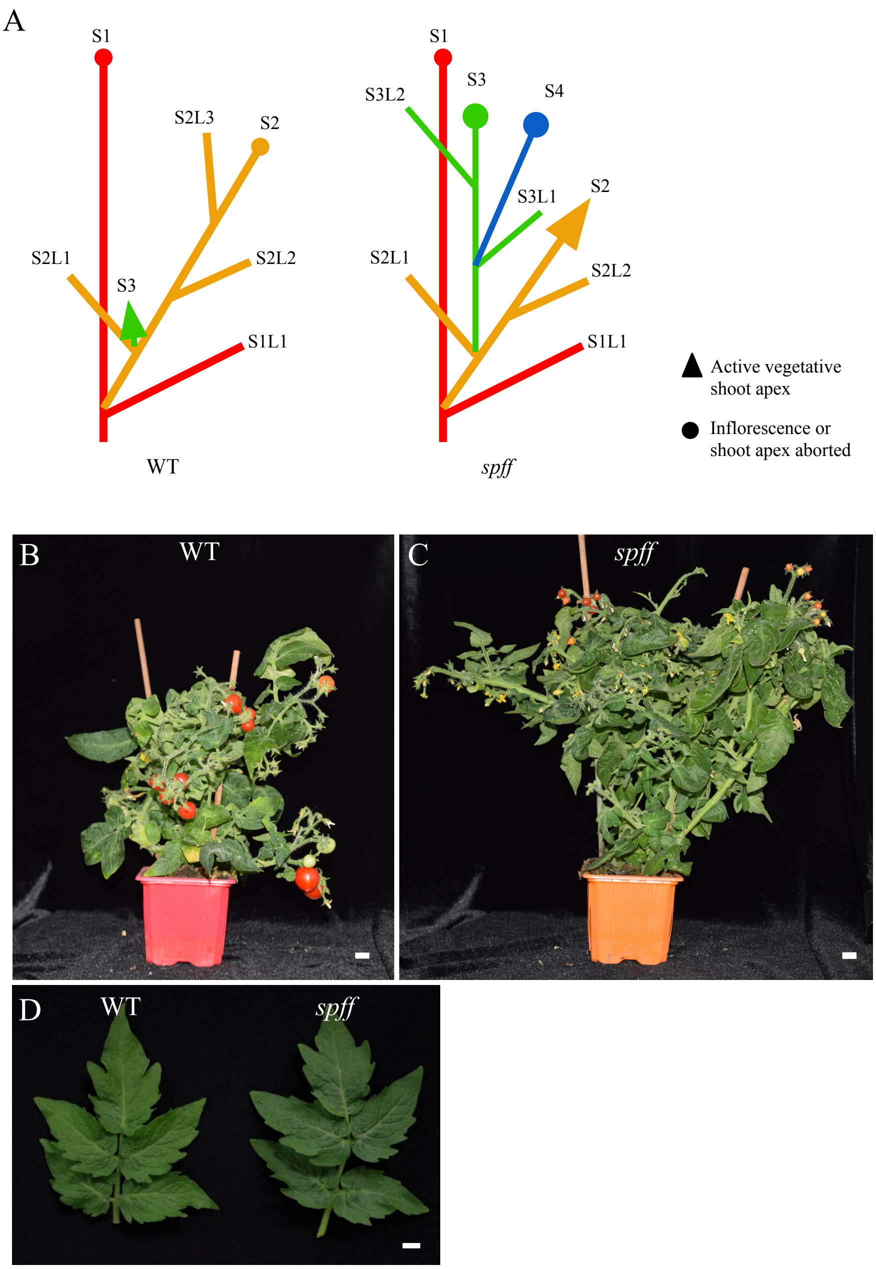

SUPPLEMENTARY FIGURE S4 | Comparison of plant branching morphology between WT and *spff* mutant. (A) Diagram depicting the branching pattern of axillary shoot and inflorescence development in WT (left) and the *spff* (right). Shoots and leaves are indicated by S and L, followed by number representing the order of emergence (e.g. S1 indicates first shoot (main stem) and S2L1 indicates first leaf of second shoot developed from axillary buds). Representative plants in WT **(B)** and *spff* mutant **(C)**. Morphology of mature leaves in WT (left) and *spff* mutant (right) **(D)**. No excess anthocyanin accumulation was observed in *spff* mutant compared to WT. Bars are 1 cm.

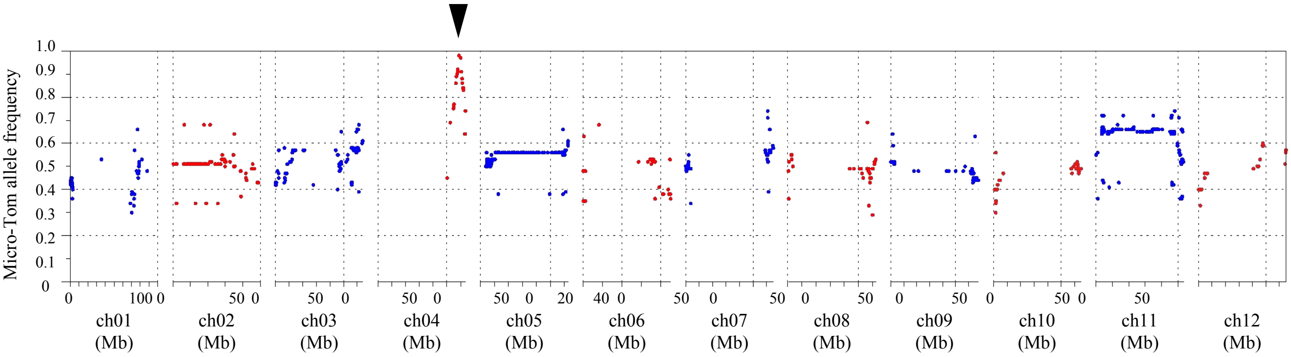

SUPPLEMENTARY FIGURE S5 | Allele frequencies of SNP markers. Scatter plots show the Micro-Tom allele frequency of each marker with genetic position in plants that exhibited *spff* mutant phenotypes at F_2 population of Micro-Tom *spff* and Ailsa Craig WT. Arrowhead indicates the peak of a high frequency cluster of Micro-Tom allele against Ailsa Craig allele.

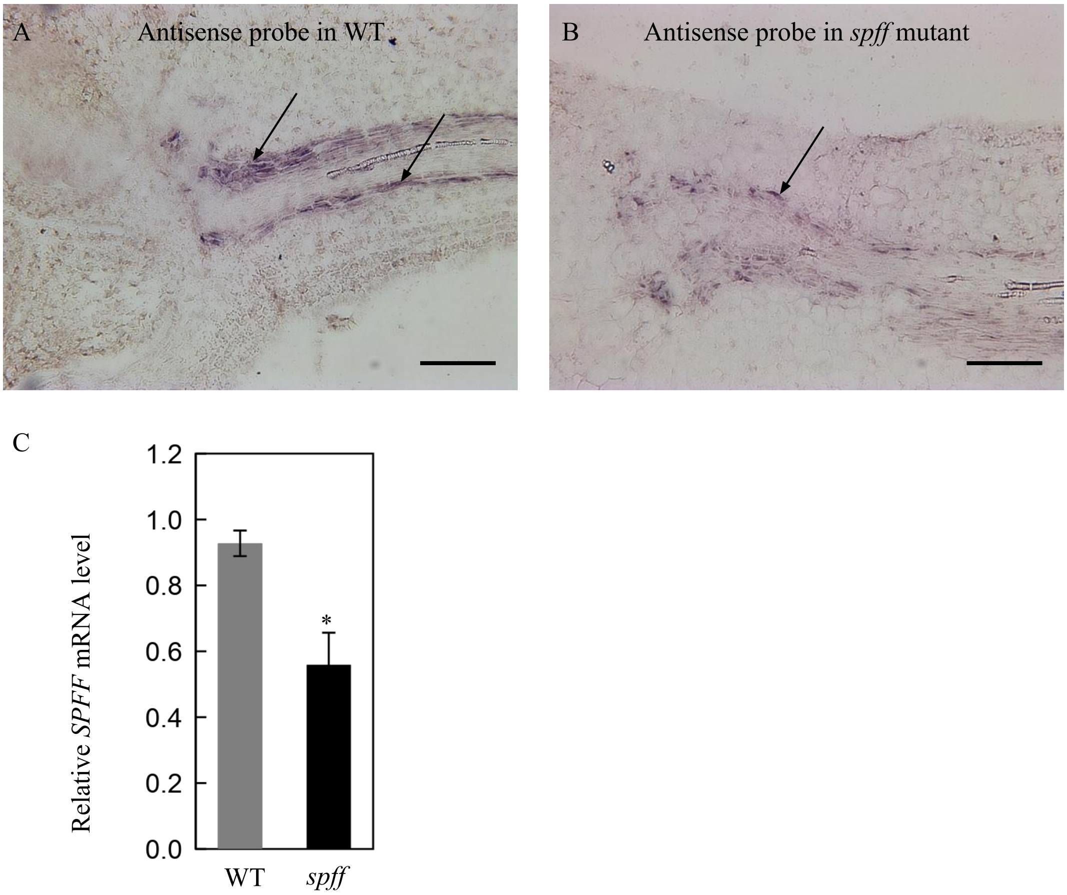

SUPPLEMENTARY FIGURE S6 |. *SPFF* mRNA level is down-regulated in the *spff* mutant. (A) *in situ* hybridization experiment to detect *SPFF* mRNA in developing buds. Floral buds size of 2.9 mm was used for the experiment as similar to **Figure 8B and E**. Distribution of *SPFF* mRNA was detected in vascular bundles by an antisense probe in WT **(A)** and *spff* mutant **(B),** although the signal of the latter was weaker than WT. Black arrows indicate signals detected in vasculature bundles and their distribution in the receptacle. The panel (A) is adopted from **Figure 8B**. Bars are 100 µm. **(C)** qRT-PCR analysis of *SPFF* mRNA level in mature leaves. The *SPFF* mRNA level was lower in the *spff* mutant compared to WT. At least three biological repetitions were performed and their mean values with SE are shown. Asterisk indicates significant difference between WT and *spff* mutant (Student *t* test, $p< 0.01$).

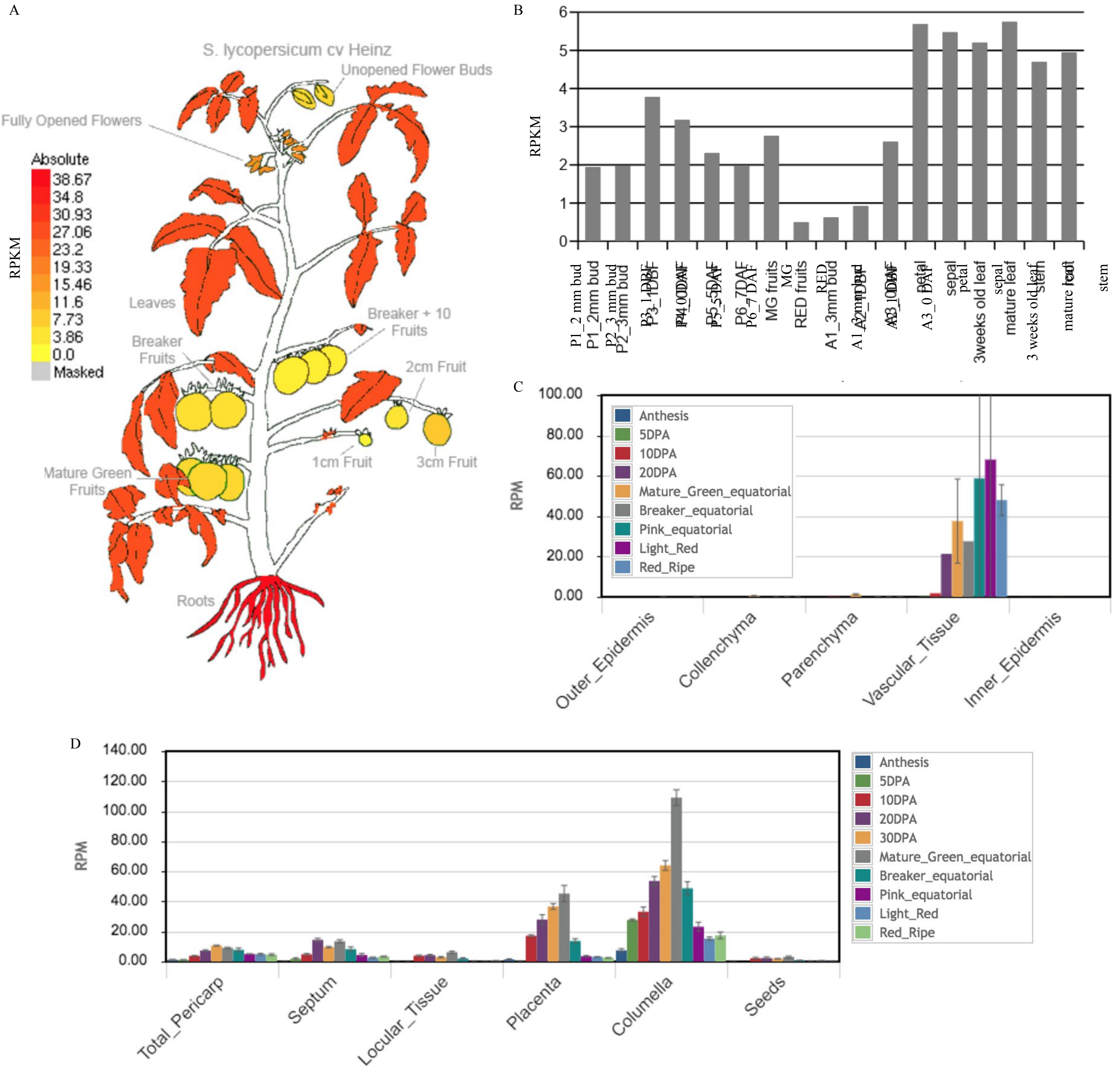

SUPPLEMENTARY FIGURE S7 | Spatiotemporal expression pattern of *SPFF* in various tomato organs, tissues and cells. **(A)** Expression images of *SPFF*, visualized in Tomato eFP browser (Winter et al., 2007), at tomato plant cv. Heinz 1706 organs (Tomato Genome Consortium, 2012. **(B)** Expression pattern of *SPFF* in organs and inflorescence tissues of cv. Micro-Tom (Ezura et al., 2017). P1-6, pistil samples; MG, mature green fruits at 33 days after flowering; RED, red fruits at 44 days after flowering; A1-3, anther samples; DBF, day before flowering; DAF, days after flowering. Fruit pericarp cell/tissue- **(C)** and fruit tissue- **(D)** and expression pattern of *SPFF*, visualized in Tomato Expression Atlas (Fernandez-Pozo et al., 2017), in cv. M82 (Shinozaki et al., 2018). DPA, days post anthesis; RPKM, reads per kilobase of transcript per million of mapped reads; RPM, reads per million of mapped reads.

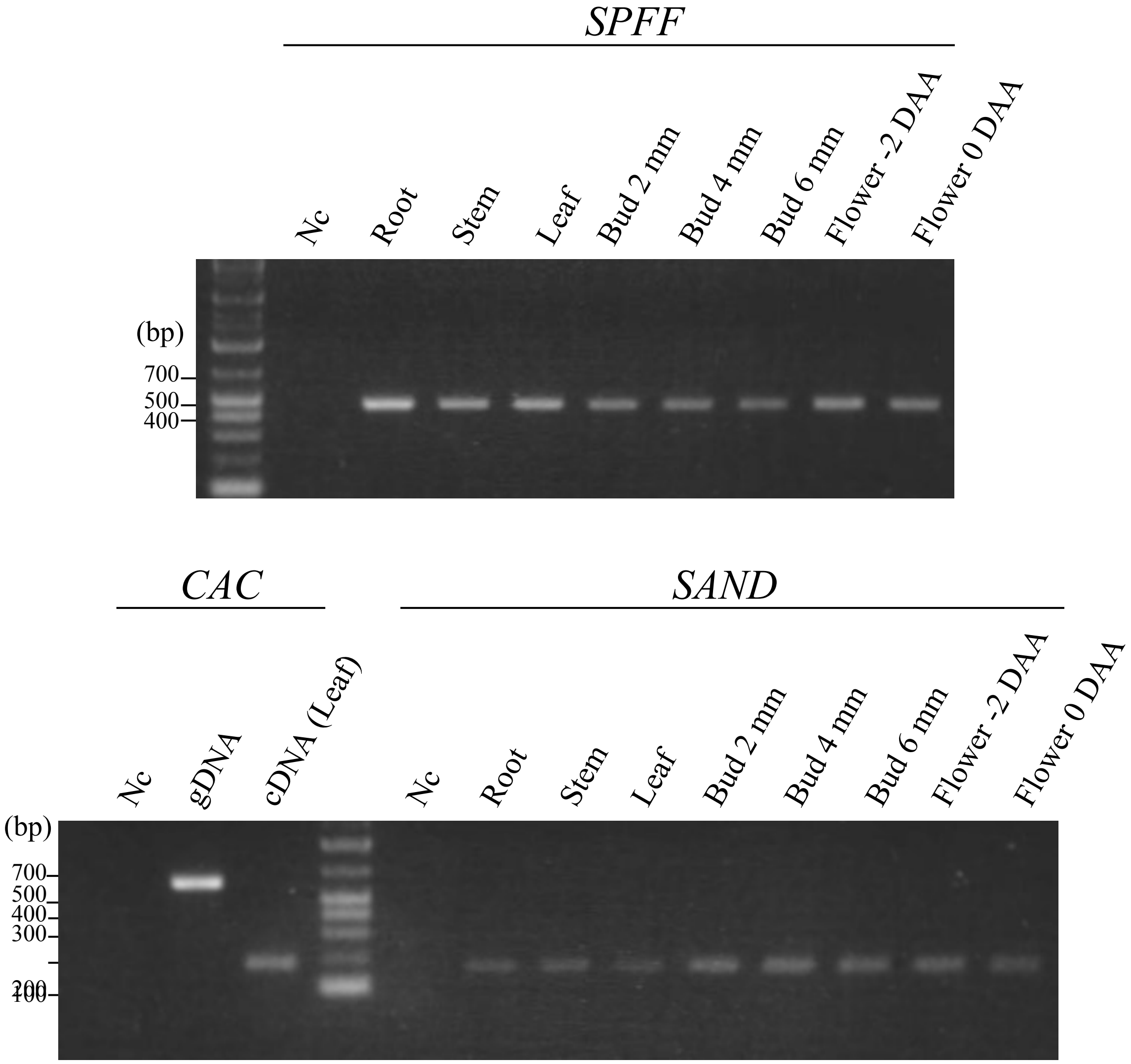

SUPPLEMENTARY FIGURE S8 | The Expressions of *SPFF* in various tissues confirmed by RT-PCR.
RT-PCR of *SPFF* was carried out with GoTaq Green Master Mix (Promega) using cDNA, genomic DNA (gDNA) or RNase-free water as a negative control (Nc). *SAND* was used as an internal control. gDNA and cDNA templates were used for *CAC* amplification to confirm the absence of gDNA contamination in cDNA. The amplicon sizes were matched in accordance with those expected by tomato gene annotation iTAG3.20 (https://solgenomics.net/organism/Solanum_lycopersicum/genome). Thermal cycling conditions were: 95°C for 3 min followed by 32 (cDNA and Nc) or 35 (gDNA) cycles of 30 s at 95°C, 30 s at 55°C and 30 s at 72°C, and finally 5 min at 72°C.

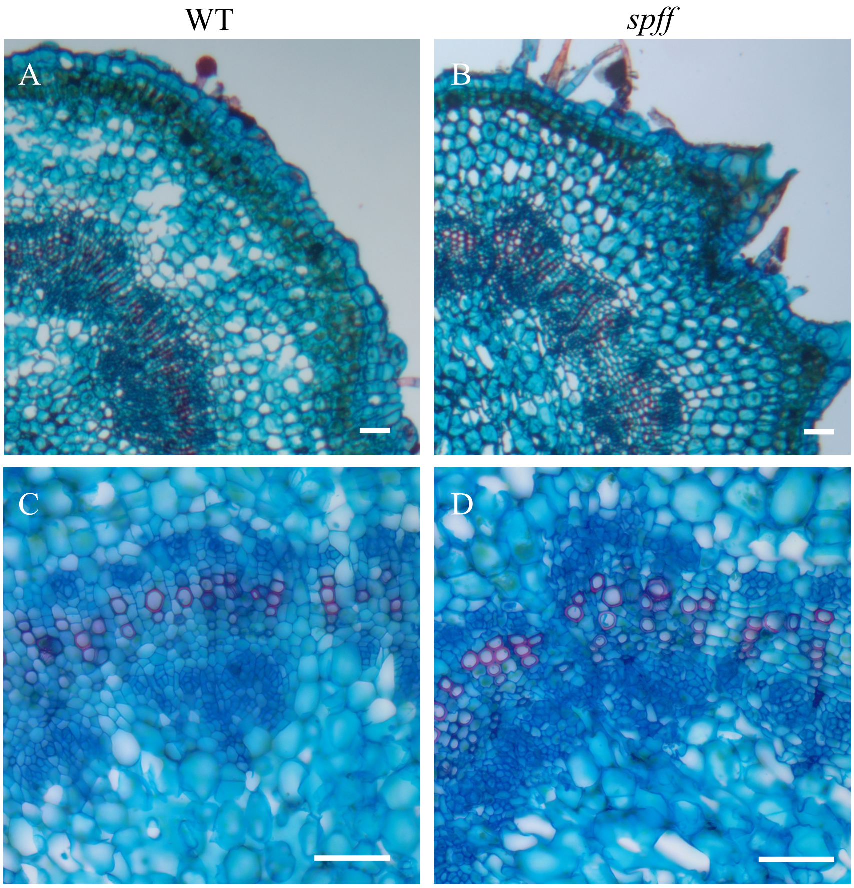

SUPPLEMENTARY FIGURE S9 | Transversal sections of *spff* receptacle. Samples were stained by Safranin O and Astra-blue double staining of DAF0 receptacle of WT **(A)(C)** and *spff* mutant **(B)(D)**. Bars are 50 μm.
